# Phytochemical Composition and Acute Hypoglycemic Effect of *Jefea lantanifolia* (S. Schauer) Strother in Rats

**DOI:** 10.3390/plants14193054

**Published:** 2025-10-02

**Authors:** Fereshteh Safavi, Sonia M. Escandón-Rivera, Adolfo Andrade-Cetto, Daniel Rosas-Ramírez

**Affiliations:** 1 Laboratorio de Etnofarmacología, Departamento de Biología Celular, Facultad de Ciencias, Universidad Nacional Autónoma de México, Av. Universidad 3000, Mexico City 04510, Mexico; 2 Departamento de Química de Biomacromoléculas, Instituto de Química, Universidad Nacional Autónoma de México, Av. Universidad 3000, Mexico City 04510, Mexico

**Keywords:** *Jefea lantanifolia* (S. Schauer) Strother, hypoglycemic effect, fasting, postprandial, phytochemical analysis, acute toxicity

## Abstract

*Jefea lantanifolia* (S. Schauer) Strother is traditionally used in Hidalgo, Mexico, to manage type 2 diabetes (T2D). The aerial parts are prepared as an infusion and consumed throughout the day. This study conducted a 2 h acute experiment under both fasting and postprandial conditions to evaluate the effects of the aqueous infusion (AE), the ethanol–water extract (EWE), and their isolated constituents in hyperglycemic rats. Structures were established using conventional spectroscopic methods. The absolute configuration was determined by optical rotation and calculated electronic circular dichroism (ECD) methods. Phytochemical analysis led to the isolation of six compounds: luteolin (**1**); 2β-hydroxy-dimerostemma brasiolide-1-*O*-(3-hydroxymethacrylate) (**2**); homoplantaginin (**3**); cynarin (**4**); luteolin-7-*O*-glucoside (**5**); and nepitrin (**6**). The extract was deemed safe at a dose of 2 g/kg b. w. in acute toxicity assays. In vivo experiments showed significant reductions in blood glucose levels during fasting, with compounds **2** and **3** achieving reductions of 42% and 40%, respectively, compared to 51% with glibenclamide. Postprandially, all treatments demonstrated effective glucose-lowering activity, particularly compound **3** and the EWE. These findings support the traditional use of *J. lantanifolia* and highlight its phytochemicals as promising candidates for further pharmacological investigation. Long-term studies and high-dose evaluations are warranted to validate therapeutic potential and establish safety profiles.

## 1. Introduction

As of 2025, an estimated one in nine individuals globally is living with diabetes. Projections by the International Diabetes Federation (IDF) indicate that this number will rise to approximately 853 million adults by 2045—equivalent to one in eight people worldwide [1]. This represents a 46% increase, with type 2 diabetes (T2D) accounting for over 90% of cases. In Mexico, it was estimated that around 14 million individuals had diabetes in 2024, including 6.7 million with undiagnosed cases [2]. The development of T2D is driven by a combination of insulin resistance and a deficit in insulin secretion from pancreatic beta cells [3]. Sustained elevations in blood glucose contribute to progressive systemic damage, affecting multiple organs and tissues. Diabetes is associated with various risk factors, including poor dietary habits, a sedentary lifestyle, obesity, and genetic predisposition [4]. Notably, the rising prevalence of obesity, particularly in developing regions, has been strongly correlated with the increase in T2D. Additional determinants such as age, ethnicity, and family history further influence the onset of the disease [4]. In a person who has not eaten for 3 to 4 h, normal blood glucose concentration is about 90 mg/dL. Following a carbohydrate-rich meal, blood glucose levels typically do not exceed 140 mg/dL unless the individual has diabetes mellitus [5].

Glucose homeostasis is essential in both fasting and postprandial states. Acute hypoglycemic effects refer to the rapid reduction in circulating blood glucose, mediated through various physiological mechanisms, including the inhibition of hepatic glucose production, stimulation of insulin secretion, and enhancement of peripheral insulin sensitivity. Certain bioactive compounds may improve cellular responsiveness to insulin, promoting more efficient glucose uptake and utilization [6]. A significant portion of the global population relies on traditional medicines for basic healthcare [7]. The World Health Organization advocates for ethnopharmacological research to preserve valuable traditional knowledge and rigorously assess the efficacy of medicinal practices rooted in indigenous and local cultures. Such studies play a vital role in bridging ancestral wisdom with modern pharmacological evidence, promoting safer and more inclusive healthcare approaches [8]. In both developing and developed countries, medicinal plants provide opportunities for treating a wide range of illnesses through complementary and alternative medicine. For centuries, various cultures have utilized medicinal plants to manage diabetes. Mexican herbal medicine is particularly rich in biodiversity, with over 300 documented plant species used for diabetes treatment [9]. This traditional knowledge is especially relevant in rural areas where access to conventional medicine may be limited [4,6]. The genus *Jefea*, belongs to the family Asteraceae, is native to Mexico and the southwestern United States [10]. *Jefea lantanifolia* (S. Schauer) Strother, a species indigenous to Hidalgo, Mexico, is traditionally employed for its medicinal properties, particularly in managing T2D. To our knowledge, this is the first study to investigate both the phytochemical profile and biological activity of this plant. The objective was to assess the efficacy of *J. lantanifolia* in controlling acute hyperglycemia in rats under fasting and postprandial conditions. The study evaluated the effects of the plant’s aqueous extract, representative of traditional infusions, alongside purified constituents derived from ethanol–water extraction. A detailed comparison of the hypoglycemic activity between the crude extract and its isolated compounds was performed, including an analysis of extraction yields. This comprehensive approach aims to deepen the understanding of *J. lantanifolia’s* pharmacological potential and support its ethnomedical use in glucose regulation.

## 2. Results

### 2.1. Ethnobotany and Plant Collection

The traditional healer Don Salomón identifies key symptoms of diabetes, including excessive thirst (polydipsia), frequent urination (polyuria), and profound fatigue. He advises individuals showing these signs to seek laboratory diagnosis from a physician. Once confirmed, Don Salomón prescribes bitter plants—based on the belief that bitterness neutralizes excess sweetness—most commonly *Calea urticifolia* (Mill.) DC. and *Jefea lantanifolia*, locally known as “Amargoso.” For *J. lantanifolia*, he recommends preparing an infusion by boiling 20 g of the dried aerial parts in 500 mL of water. This infusion should be consumed ad libitum throughout the day, similar to ‘agua de uso.’ He suggests a 15-day treatment period followed by a one-week break, cautioning that excessive intake may cause dizziness due to its perceived potency. In July 2022, aerial parts of *J. lantanifolia* were collected in the town of Tamla (coordinates: 21°00′605″ N, 98°47′966″ W) in the state of Hidalgo, Mexico. Botanical authentication was conducted by M. en C. Ramiro Cruz Durán, and a voucher specimen (ETNOF261) was deposited in the Herbarium of the Faculty of Science.

### 2.2. Dose Calculation for Extracts and Compounds

Based on the yield of the extracts (Section 2.3), the equivalent doses of AE and EWE were calculated to be 364 mg/kg b.w. and 348 mg/kg b.w., respectively. Subsequently, the doses of compound **2**, compound **3**, and compound **4** purified from the AE extract were determined to be 0.58 mg/kg b.w., 0.27 mg/kg b.w., and 1.7 mg/kg b.w., respectively. The extracts and compounds were dissolved in a 10 mg/kg b.w. physiological solution for in vivo testing.

### 2.3. Acute Oral Toxicity Test

A toxicity study was conducted on mice administered 2000 mg/kg b.w. of the AE to assess its safety. The animals were monitored for 14 days for signs of toxicity or adverse effects. No significant toxic effects were observed, indicating that the extract is safe at this dosage. This suggests that *J. lantanifolia* can be used safely at therapeutic doses without a significant risk of acute toxicity. It is important to note that many in vivo tests on aqueous extracts support the safety of herbal medicines. In contrast, most in vitro tests on isolated single cells, particularly those using non-aqueous extracts, yield contradictory results, fueling the ongoing debate about the safety of herbal medicines. Toxicity studies on herbal medicines should align with their traditional use to facilitate rational discussions about their safety and potential benefits. While no single study definitively concludes that the safety of crude extracts alone justifies human extrapolation, the sources listed collectively highlight the complexities and necessary considerations. They emphasize the need for a comprehensive safety assessment that includes identifying active compounds, understanding bioavailability, and accounting for species-specific differences before extrapolating animal data to humans [11,12].

### 2.4. HPLC/DAD Profiles

**Figure 1:**
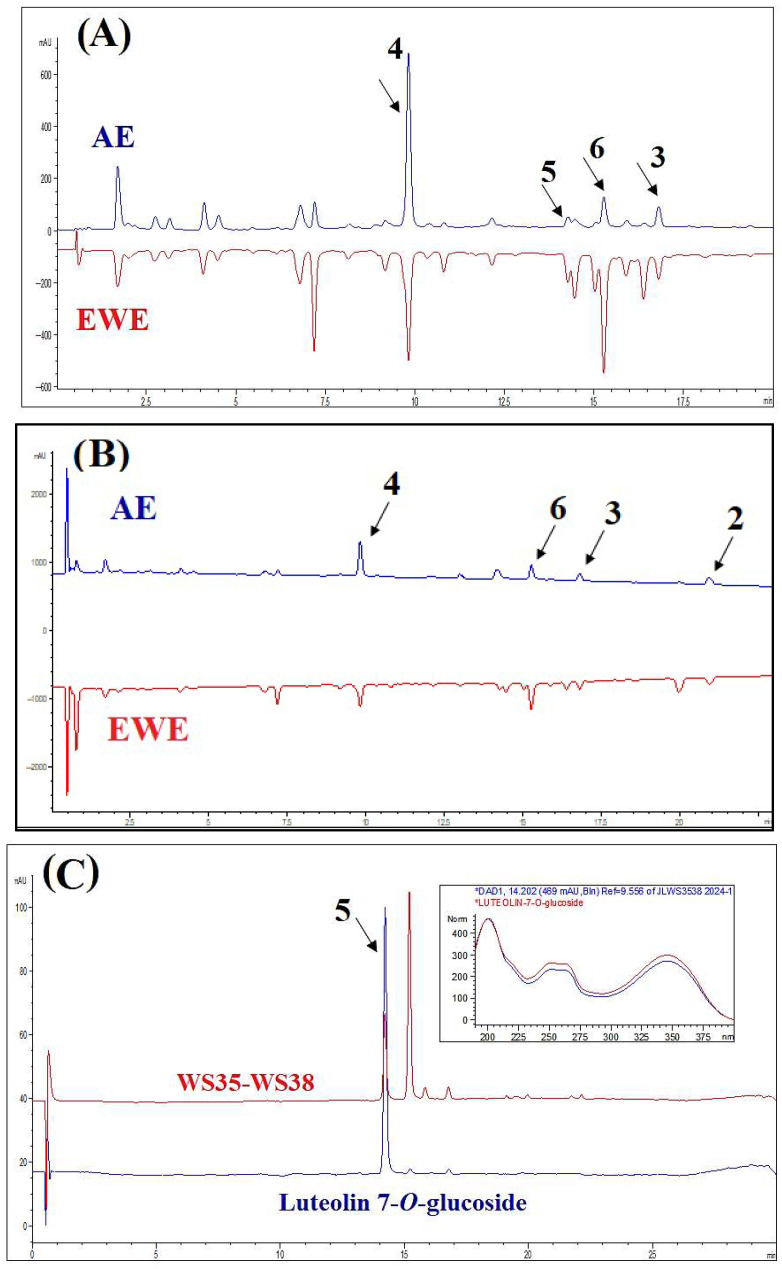
HPLC-DAD profiles of the aqueous extract (AE) and ethanol–water extract (EWE), as well as purified compounds, at 320 nm (**A**) and 210 nm (**B**). Panel (**C**) displays the chromatogram of luteolin-7-*O*-glucoside and subfractions WS35-WS38. The insert shows the UV spectra (200–400 nm) of the representative peak alongside the standard spectrum of luteolin-7-*O*-glucoside, which match closely.

Typical HPLC chromatograms of AE and EWE, along with their major components, are presented in Figure 1. Most components in the chromatographic profiles exhibited absorbance maxima at 320 nm and 210 nm, and EWE displayed a similar phytochemical profile to AE. For further fractionation and isolation of the compounds, we chose AE as the traditional extract form. The main constituents of the two extracts were isolated and purified from the AE extract. Compound **1** could not be clearly identified in the chromatographic profiles due to its low concentration in the extracts. Compound **5** was identified by comparing the retention time and UV spectrum of the standard peak of luteolin-7-*O*-glucoside (>98% HPLC; ChemFaces Biochemical Co., Wuhan, China) with the corresponding peak in the chromatogram of one of the subfractions of WS.

### 2.5. Structure Elucidation of the Pure Compounds

**Figure 2:**
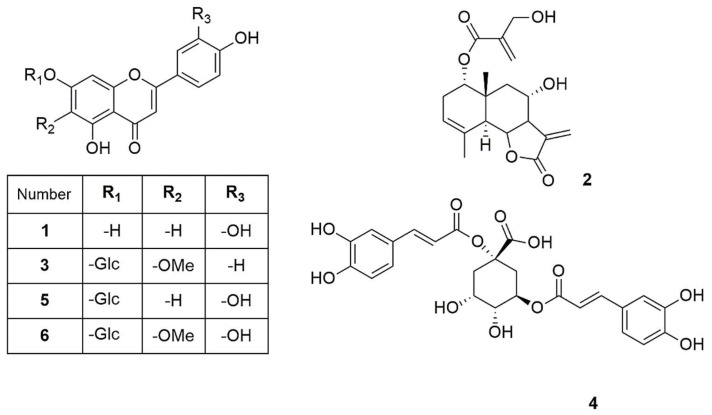
Compounds isolated from *J. lantanifolia*: Luteolin (**1**), 2*β*-Hydroxy-dimerostemma brasiolide-1-*O*-(3-hydroxymethacrylate) (**2**), Homoplantaginin (**3**), Cynarin (**4**), Luteolin-7-*O*-glucoside (**5**), Nepitrin (**6**).

**Figure 3:**
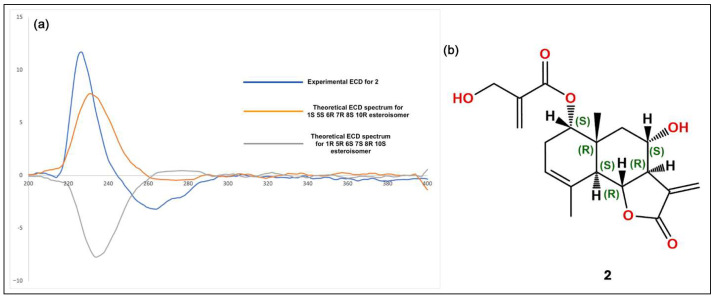
(**a**): Experimental circular dichroism (CD) spectra for compound **2** (blue line) alongside the calculated CD spectra (orange line). (**b**): Structural stereoisomer obtained for compound **2**.

The isolated compounds (Figure 2) were characterized using various spectroscopic methods, including NMR spectroscopy (^1^H and ^13^C), DEPT, HSQC, HMBC, COSY, NOESY, and TOCSY, as well as high-resolution ESI-MS, circular dichroism, and optical rotation. Based on the ^1^H-NMR data (Table 1) and the ESI-MS spectrum (Figure S24) showing 285.03746 [M-H]^−^ (calcd. for C_15_H_9_O_6_, 285.04046), compound **1** was identified as luteolin [13]. Compound **2** was obtained as a yellowish oil with the molecular formula C_19_H_24_O_6_, as deduced from NMR spectroscopy data and the HRESI-MS ion at 383.1203 [M+Cl]^−^ (calcd. for C_19_H_24_O_6_Cl^−^, 383.1272), indicating eight degrees of unsaturation (Figures S1–S9). The ^1^H, ^13^C, and DEPT spectra (Table 2; Figures S2–S4) revealed a 19-carbon unit with two methyl, five methylene, six methine, eight vinyl, and six quaternary carbons. The ^1^H and ^13^C NMR spectra (Table 2, Figures S2 and S3), HSQC (Figure S5), and COSY correlations (Figure S6) indicated a eudesmanolide skeleton containing a Δ3,4 double bond, with an allylic methyl group at 23.8/1.90 (d, *J* = 1.93 Hz; C/H-15) and another quaternary methyl group at δ_C/H_ 18.5/0.96 (s; C/H-14) as part of the backbone. The presence of a lactone ring in the molecule was suggested by the ^13^C NMR shift at δ_C_ 170.5 (C-12) and the typical ^1^H NMR shift at δ_H_ 3.97 (dd, *J* = 11.69 and 10.74 Hz, H-6) of the lactone carbonyl group. Additionally, an exocyclic olefinic methylene group at δ_C/H_ 120.2/6.16 (d, *J* = 3.38 Hz, C/H 13a) and 5.97 (d, *J* = 3.0 Hz, C/H 13b) indicated the characteristic α-methylene-γ-lactone, with their coupling greater than 3.0 suggesting a *trans*-lactone configuration [14]. A 3-(hydroxymethyl)acrylate radical was identified at position C-1 of the molecule, supported by HMBC correlations (Figure S7) showing a shift at δ_H_ 4.76 (d, *J* = 4.62 Hz, H-1) with δ_C_ 165.9 (C-1′). This carbonyl also exhibited HMBC correlations with δ_H_ 4.33 (brs, H-3′), δ_H_ 6.24 (d, *J* = 1.14, H-4′a), and δ_H_ 5.86 (d, *J* = 1.31 Hz, H-4′b). The relative configuration of compound **2** was determined using the NOESY spectrum (Table 2; Figure S8), which revealed key NOESY correlations of H-1 with H-14, H-2*β* and H-9, indicating its beta position. Additionally, H-5 was associated with H-7 and H-9α, suggesting a *trans*-type eudesmanolide skeletal configuration. To ascertain the absolute configuration of compound **2**, we measured the specific rotation and compared the experimental and calculated ECD curves for the *trans* eudesmanolide isomer (Figure 3a). The specific rotation ([α]20D = +100) was a positive value. The experimental ECD spectrum (blue line, Figure 3a) displayed a positive and negative Cotton effect at 226 nm (Δε = +11.7) and 264 nm (Δε = −3.19), respectively. This pattern was consistent with the theoretical ECD spectrum simulated for the *trans*-eudesmanolide isomer (orange line, Figure 3a). The absolute configuration of compound **2** was determined to be the 1S 5S 6R 7R 8S 10R stereoisomer (Figure 3b). This compound was identified as 2*β*-hydroxy-dimerostemma brasiolide-1-*O*-(3-hydroxymethacrylate), isolated for the first time from *Dimerostemma brasilianum* [15]. Compound **3** was obtained as a yellow powder with the molecular formula C_22_H_22_O_11_, as deduced from the NMR spectroscopy data (Table 1, Figures S10–S17) and the HRESI-MS ion at 461.1032 [M-H]^−^ (calcd. for C_22_H_21_O_11_, 461.1089), indicating twelve degrees of unsaturation. The ^1^H-NMR and ^13^C-NMR spectra, along with 2D-NMR experiments (COSY, HSQC, HMBC, NOESY, TOCSY; 400 MHz, CD_3_OD; Figures S13–S17), indicated the presence of a flavonoid with glucose and methyl moieties. The ^1^H-NMR spectrum (Figure S10) exhibited an AA′BB′ system for protons in the B-ring at δ_H_ 7.86 (d, *J* = 8.70 Hz, H-2′ and H-6′) and at δ_H_ 6.86 (d, *J* = 8.80 Hz, H-3′ and H-5′). Additionally, it featured two singlets at δ_H_ 6.62 (s, H-3) and 6.96 (s, H-8), as well as characteristic signals for the anomeric proton at δ_H_ 5.13 (d, *J* = 7.35 Hz, H-1″) for glucose and for the methoxyl group at δ_H_ 3.89 (s, OCH_3_). The position of the methoxyl was determined using the HMBC spectrum (Figure S15), which revealed a correlation between δ_H_ 3.89 and δC 134.3 (C-6). Finally, the NOESY spectrum (Figure S16) indicated an interaction between H-8 and the anomeric proton H-1″. Compound **3** was identified as homoplantaginin, which was isolated for the first time from *Arnica montana* [16]. Compound **6** was obtained as an oily yellow substance with the molecular formula C_22_H_22_O_12_, as determined by ^1^H NMR (400 MHz, DMSO-D_6_) and HRESI-MS (Figure S17), which showed ions at 479.1271 [M+H]^+^ (calcd. for C_22_H_23_O_12_, 479.1195). Its spectral data were similar to that of compound **3,** with H-2 appearing at δ_H_ 6.66 (s) and H-8 at δ_H_ 6.96 (s). However, in the B ring, it exhibited an ABX system with δ_H_ 6.77 (d, *J* = 8.40 Hz, H-3′), δ_H_ 7.36 (d, *J* = 2.36 Hz, H-6′), and δ_H_ 7.41 (dd, *J* = 8.44 Hz and 2.39 Hz, H-2′), indicating that the only difference from compound **3** is the presence of a hydroxyl group at C-4′. Compound **6** was identified as nepitrin, which was isolated for the first time from *Nepeto hindostana* [17]. Compound **4** was obtained as a white powder with the molecular formula C_25_H_24_O_12_, as determined from NMR spectroscopy data and the HRESI-MS ion at 515.1266 [M-H]^−^ (calcd. for C_25_H_23_O_12_, 515.1195), indicating 13 degrees of unsaturation (Figures S18–S23). The ^1^H NMR, ^13^C NMR, and DEPT spectra, along with 2D NMR experiments (COSY and HMBC; 400 MHz, D_2_O; Table 3; Figures S22–S23), suggest the presence of *di*-caffeoylquinic acid. The DEPT, ^1^H-NMR, and ^13^C-NMR spectra (Figures S19–S21) revealed typical signals for quinic acid, including two methylenes at δ_C/H_ = 30.7/2.33 (dd, *J* = 16.48, 2.87 Hz, C/H-2a) and 30.7/3.05 (dd, *J* = 16.37, 2.60 Hz, C/H-2b), δC/H = 40.8/1.79 (dd, *J* = 13.58, 11.42 Hz, C/H-6a), and δC/H = 40.8/2.46 (dt, *J* = 13.25, 3.65 Hz, C/H-6b). Additionally, three oxygenated methine signals were observed at δ_C/H_ = 73.3/5.27 (m, C/H-3), 73.6/3.80 (dd, *J* = 9.88, 3.62, C/H-4), and 66.9/4.38 (td, *J* = 11.4, 4.56, C/H-5); a quaternary carbon atom at δC = 82.6 (C-1); and a carbonyl carbon at δC = 177.5 (C-7). Furthermore, two *trans*-caffeoyl moieties with characteristic AX systems were observed at δ_C/H_ = 146.8, 146.3/7.45, 7.33 (d, *J* = 15.98, C/H-7′; d, *J* = 16.00, C/H-7″) and δ_C/H_ = 114.3, 115.1/6.16, 6.27 (d, *J* = 15.97, C/H-8′; d, *J* = 15.97, C/H-8″). The ^13^C NMR spectrum indicates a shift in C-1 (82.6), suggesting a ramification at the C-1 position, while the HMBC NMR spectrum (Figure S23) showed an interaction between H-3 and carbonyl C9″. These results indicate that the caffeoyl moieties are connected at positions 1 and 3 of quinic acid. Therefore, Compound **4** was identified as to be 1,3-*di*-caffeoylquinic acid (cynarin), and the NMR data were compared to previously published results [18].

### 2.6. Acute Hypoglycemic Effect in the Fasting State

**Figure 4:**
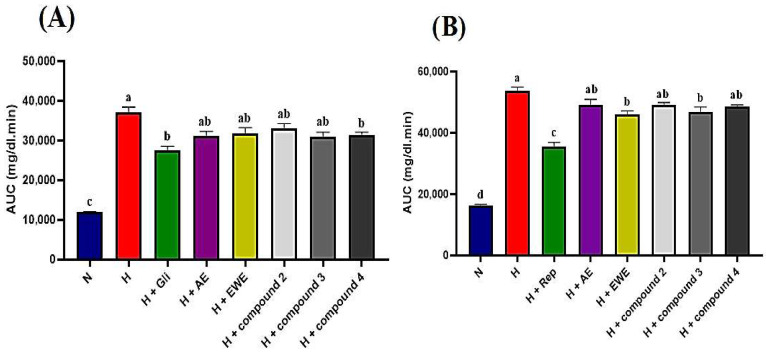
(**A**,**B**): Area under the curve (AUC) for glucose levels derived from normal and glucose response curves. Statistically significant differences are indicated by different letters (a > b > c > d). Group designations are as follows: N (normal control); H (hyperglycemic control); H + Gli (hyperglycemic group treated with Glibenclamide); H + Rep (hyperglycemic group treated with Repaglinide); H + AE (hyperglycemic group treated with aqueous extract of *J. lantanifolia*); H + EWE (hyperglycemic group treated with ethanol–water (1:1) extract of *J. lantanifolia*); H + compound **2** (hyperglycemic group treated with 2*β*-Hydroxy-dimerostemma brasiolide-1-*O*-(3-hydroxymethacrylate)); H + compound **3** (hyperglycemic group treated with homoplantaginin); H + compound **4** (hyperglycemic group treated with cynarin). Data are expressed as mean ± SEM, *p* < 0.05; n = 6.

STZ-NA-induced diabetic rats were administered AE, EWE, compound **2**, compound **3**, and compound **4** over a two-hour period, with blood glucose levels measured at 30 min intervals. Table 4 summarizes the blood glucose levels (mg/dL) measured at various post-treatment times. In the normal control group, blood glucose levels remained stable, ranging from 98 to 101 mg/dL throughout the tests. In contrast, the hyperglycemic control rats showed no significant reduction, with glucose levels consistently at 298 mg/dL after 120 min. As expected, glibenclamide (5 mg/kg b.w.) significantly reduced glucose levels to 151 mg/dL after 120 min (*p* < 0.05). Glibenclamide exhibited a hypoglycemic effect, with blood glucose levels decreasing after 30 min compared to the hyperglycemic control group. A further reduction was observed after 60 min relative to baseline (T0), achieving the maximum effect at 120 min, where blood glucose levels were lowered by 156 mg/dL. Data presented in Table 4 indicate that both the extracts and the tested compounds exhibited hypoglycemic effects compared to the hyperglycemic control group (*p* < 0.05). The observed reductions began at 60 min for AE, compound **2**, and compound **4**, while EWE and compound **3** showed reductions after 30 min, which persisted throughout the test relative to T0. AE, EWE, compound **2**, compound **3**, and compound **4** lowered blood glucose levels by 100, 102, 142, 131, and 122 mg/dL, respectively, at 120 min. However, the AUC analysis provides a more comprehensive comparison. Figure 4A shows that glibenclamide reduced blood glucose levels by 26%, while AE, compound **3**, and compound **4** reduced levels by 16%, and EWE and compound **2** reduced levels by 14% and 11%, respectively. Compound **4** and glibenclamide received the same designation letter, indicating no statistically significant difference in their effects (*p* > 0.05) (Figure 4A). This suggests that both treatments had similar overall efficacy under the conditions of this study. The positive control, however, demonstrated a larger reduction in blood glucose levels, lowering them by 156 mg/dL.

### 2.7. Acute Hypoglycemic Effect in the Postprandial State

In the postprandial state, glucose levels in the normal control group increased significantly, peaking after 30 min, showing a 53% rise before returning to baseline after 120 min. In contrast, the hyperglycemic control group exhibited severe hyperglycemia, reaching a 75% increase at 30 min, with glucose levels remaining significantly elevated compared to the normal control group even after 120 min. In the repaglinide-treated group (1 mg/kg), a delayed rise in blood glucose levels was observed. At 30 min, maximum plasma glucose levels reached 350 mg/dL, reflecting a 12% increase, before beginning to decline, with a maximum decrease of 92 mg/dL noted after 120 min (Table 5). As shown in Table 5, all tested samples demonstrated a blood glucose-lowering effect in the postprandial state, with no significant differences observed between each group and its respective initial values (T0) after 120 min. Notably, compound **3** and EWE emerged as the most effective treatments, yielding the highest reductions in postprandial blood glucose and AUC (Table 5, Figure 4B).

## 3. Discussion

T2D is a rapidly growing, multifactorial metabolic disease that affects millions of people worldwide [2]. This chronic condition is commonly associated with hyperglycemia and comorbidities such as obesity and hypertension [19]. In the Mexican town of Tamala, a plant specialist named Don Salomon uses *J. lantanifolia* to treat hyperglycemia in patients with type 2 diabetes. This study reports positive results from using crude extracts and selective major constituents of *J. lantanifolia* to control pre- and postprandial blood glucose levels in STZ-NA-induced hyperglycemic rats for the first time. It is important to note that the STZ-NA model does not fully capture all the features of T2D, particularly insulin resistance. However, the diminished glucose tolerance observed in these organisms is primarily attributed to reduced insulin secretion from the remaining functional *β*-cells [20], making it a useful model for testing hypoglycemic compounds [21].

Potentiation or synergy occurs when the combined effect of constituents exceeds the expected additive effect. In additive combinations, the result is Acute oral toxicity testing confirmed that the AE extract was safe at its traditional dose, making it suitable for in vivo pharmacological tests. However, demonstrating and defining a combination effect is challenging due to the complex methodology required to identify interaction effects. Thus, it is essential to assess the activity of individual compounds and compare them to the corresponding dose of the mixture [22]. Administration of AE, EWE, and the pure compounds to hyperglycemic rats resulted in a decrease in fasting blood glucose levels two hours post-treatment. Notably, apart from compounds **2** and **3**, which reduced fasting blood glucose levels by 42% and 40%, respectively (approximately 10% lower than the 51% reduction observed in the positive control), the other samples exhibited similar effects with an average reduction of 34% (Table 4). Ultimately, the overall activity of medicinal plant extracts derives from multiple compounds that may have synergistic, additive/non-interactive, or antagonistic effects [23]. A pure summation of the compounds’ effects occurs when their interactions are additive. In contrast, antagonistic interactions lead to effects that are less than additive, meaning the outcome is smaller than the sum of the individual components [24]. This phenomenon appears to be evident in our study, both in fasting and postprandial states. For instance, Figure 4B demonstrates that compound **3** and the EWE, identified as the most effective treatments in this context, are statistically different from the other treatment groups. Although precise quantitative measurements of the compounds in the two extracts were not conducted in this study, Figure 1A suggests that the difference in the amount of compound **3** between the AE and EWE is minimal. Instead, the higher concentrations of compounds **4** and **6** in the AE may be responsible for the reduced activity of the AE during the antagonistic interaction observed in the postprandial state.

An HPLC-DAD method was developed to qualitatively identify the compounds isolated from the aqueous extract and to compare them with the profile of the ethanol–water extract. Some metabolites were easily identified by comparing their retention times and UV spectra with reference standards, while others could not be identified due to low concentrations in the extracts. Although this methodology is highly sensitive and allows for a complete chromatogram of the extract or standards, it does not identify all traces as effectively as other techniques, such as LC-MS/MS, which can provide more comprehensive information on the metabolic content of the extracts. Both techniques can be affected by matrix effects that may suppress or enhance analyte signals, particularly at low concentrations or when sample preparation is less reproducible. These limitations can lead to inaccurate quantification of trace bioactive compounds, potentially distorting the interpretation of their biological effects—either underestimating or overestimating their relevance or toxicity. Therefore, rigorous sample preparation and, when possible, advanced detection methods like mass spectrometry should be prioritized for sensitive and reliable trace analysis [25].

In this study, four flavonoids from AE were identified, enhancing the characterization of its bioactive properties: luteolin, luteolin-7-*O*-glycoside, homoplantaginin (hispidulin-7-glucoside), and nepitrin (nepetin-7-glucoside). Flavonoids are recognized for their anti-inflammatory, antioxidant, and hypoglycemic properties. Numerous in vivo and in vitro studies have demonstrated the blood glucose-lowering effects of plant flavonoids [26]. These compounds effectively regulate blood glucose levels in diabetic rats treated with streptozotocin and help prevent diabetes-related complications. Flavonoids exert hypoglycemic effects by inhibiting glucose synthesis, enhancing glucose uptake in muscle tissue, increasing insulin secretion, decreasing insulin resistance, and promoting pancreatic beta cell proliferation while reducing apoptosis [27]. Luteolin and luteolin-7-*O*-glucoside are well-known flavonoids that have been identified and purified from various plant genera. They exhibit significant biological activities that support their potential therapeutic applications, particularly in diabetes and oxidative stress.

Extensive evidence highlights the therapeutic benefits of luteolin, including its anti-inflammatory, antioxidant, and blood sugar-lowering properties, emphasizing its potential as a multifunctional therapeutic agent. Both luteolin and luteolin-7-*O*-glucoside demonstrate impressive inhibition of α-glucosidase, surpassing that of acarbose, a common hypoglycemic drug [28]. This suggests that these compounds may effectively lower postprandial blood glucose levels. Additionally, luteolin-7-*O*-glucoside has shown significant hypoglycemic effects in both in vitro and in vivo studies, inhibiting α-glucosidase and α-amylase, which leads to lower blood glucose levels in diabetic models [29]. Further evidence supports its potential to prevent diabetes complications by lowering plasma glucose concentrations and improving lipid profiles [30]. Luteolin-7-*O*-glucoside, a major compound in traditional Chinese green tea, exerts a regulatory effect on glucose metabolism by activating the AMPK pathway. This activation leads to an increased p-AMPK/AMPK ratio and a reduction in the expression of hepatic gluconeogenic enzymes, including G6Pase and PEPCK [31].

Nepitrin has been extensively studied for its anti-inflammatory and anti-arthritic properties [32,33,34]. However, due to the limited quantity of compound obtained during purification, in vivo studies were not feasible. As one of the major components of AE, homoplantaginin exhibits a wide range of biological activities, including anti-inflammatory and antioxidant effects [35,36]. Previous studies have shown that homoplantaginin inhibits inflammatory signaling pathways involving Toll-like receptor 4 and nucleotide-binding domain-like receptor 3, thereby alleviating palmitic acid-related damage to vascular endothelial cells [37]. Homoplantaginin (0.1, 1, and 10 μM) activates the AMPK/mTORC1/TFEB signaling pathways, repairs lysosomal function by upregulating LAMP1 and CTSB expression, and subsequently mitigates vascular endothelium damage in db/db mice by inhibiting high glucose-induced apoptosis through enhanced autophagy [38]. Homoplantaginin is one of the major compounds found in the leaf extract of *Abrus precatorius*. This extract may enhance insulin sensitivity in skeletal muscles and reduce hyperglycemia by increasing insulin-stimulated glucose uptake and the expression of insulin receptor substrate 1 and Akt substrate of 160 kDa [39]. In this study, we evaluated the acute hypoglycemic effect of homoplantaginin for the first time. As noted earlier, this compound demonstrated one of the most significant blood glucose-lowering effects, particularly during fasting (Table 4).

The bioavailability, pharmacokinetics, and metabolism of flavonoids have been studied to assess their crucial role in the chemoprevention of diseases such as diabetes. By analyzing the pharmacokinetics of luteolin, luteolin-7-*O*-glucoside, and homoplantaginin based on previous studies, we can draw conclusions about their potential roles in mediating acute hypoglycemic effects in hyperglycemic rat models. Luteolin is known for its rapid absorption and significant first-pass metabolism, resulting in a bioavailability of approximately 4.10%. This compound effectively reaches systemic circulation as various glucuronides and has demonstrated considerable efficacy in lowering blood glucose levels, particularly in fasting states, by enhancing insulin sensitivity and modulating glucose uptake in peripheral tissues. In postprandial conditions, luteolin can inhibit glucose spikes by interfering with carbohydrate-digesting enzymes, further establishing its role as a potent hypoglycemic agent. In contrast, luteolin-7-*O*-glucoside is absorbed only after being converted to luteolin, which may limit its direct hypoglycemic effects. While it has shown improvements in metabolic parameters in diabetic models, its overall potency in reducing blood glucose levels is less pronounced than that of luteolin [40,41]. In a study, homoplantaginin exhibited a unique profile with rapid absorption, achieving a mean Cmax of 0.77 to 1.27 nmol/mL; however, its absolute oral bioavailability was only 0.75%. Given the compound’s effectiveness in the postprandial state in our research, its rapid absorption and apparent passive transport mechanism in the intestine suggest that, despite its low absolute oral bioavailability, the amount reaching circulation may still be sufficient to significantly influence glucose metabolism after meals. Nevertheless, further investigation is needed to clarify its contribution to the hypoglycemic activity of the total extract [42]. Additionally, there is a lack of specific scientific literature examining the pharmacokinetics of compounds **2**, **4**, and **6**, highlighting a significant gap in understanding how these compounds may influence the overall pharmacokinetic profile of the total extract.

A broad range of biological activities and structural diversity makes sesquiterpene lactones particularly interesting for pharmacological applications [43]. They are recognized for their significant anti-inflammatory, hypoglycemic, and hypolipidemic properties, which make them suitable for the treatment of metabolic syndrome [44]. In this study, we successfully purified and elucidated the structure of 2*β*-Hydroxy-dimerostemma brasiolide-1-*O*-(3-hydroxymethacrylate) (compound **2**), identified as a *trans*-6,7-eudesmanolide sesquiterpene lactone. This compound was previously isolated from the diethyl ether: petroleum ether (1:2) extract of *Dimerostemma brasilianum* [15], making our study a second attempt to purify and characterize it. For molecular structure elucidation, we employed comprehensive one- and two-dimensional NMR techniques alongside ESI-MS, unlike the previous study, which lacked thorough spectral data. Additionally, optical rotation and CD spectroscopy were utilized to confirm the stereochemistry of the compound. Since its first purification in 1982, no literature has documented the pharmacological or biological effects of this compound, including its hypoglycemic effects or potential applications in other diseases. Our study is the first to investigate the pharmacological properties of this compound, highlighting its acute hypoglycemic effect, which represents a significant advancement in its pharmacological investigation. Cynarin, a well-known compound derived from the artichoke (*Cynara scolymus* L.), has garnered attention for its potential health benefits, particularly regarding fatty liver disease and diabetes. It has been shown to inhibit α-glucosidase, which may reduce glucose uptake in the intestine and lower postprandial blood glucose levels [45]. Artichoke water extracts containing cynarin have demonstrated improvements in glucose uptake and utilization in insulin-resistant HepG2 cells, alongside significant reductions in fasting blood glucose levels and enhanced lipid profiles in streptozotocin-induced diabetic rats. Thus, cynarin may help alleviate hyperglycemia and dyslipidemia associated with diabetes [46,47].

Overall, these findings highlight the significance of the traditional dosage of the infusion extract and its key components as promising candidates for further research and clinical applications in diabetes treatment. However, it is essential to establish the long-term therapeutic efficacy of the extract and its compounds. Additionally, further studies are necessary to confirm the potential benefits of higher concentrations of these compounds and to evaluate the risks associated with increased dosages.

## 4. Materials and Methods

### 4.1. Ethnobotany and Plant Material

Several direct interviews were conducted with healer Salomón Villegas between 2021 and 2024. He lives and works in Tamala, in the Mexican state of Hidalgo where diabetics from nearby regions regularly seek his advice on diabetes treatment. Basic information about the plant was collected, including the parts used, methods of preparation, administration, dosage, collection, and storage. Additionally, details regarding the healer’s knowledge of diabetes were gathered. All data were collected with the healer’s permission and informed consent, following the directives of the International Society of Ethnobiology [48].

### 4.2. Experimental Techniques

The NMR spectroscopic data were acquired in methanol-d_4_ (CD_3_OD), chloroform-d (CDCl_3_), deuterated oxide (D_2_O), and dimethyl sulfoxide-D_6_ (DMSO-D_6_) (99.95%, Sigma-Aldrich, Milan, Italy). All one- and two-dimensional NMR spectra were recorded on a Bruker Avance III spectrometer operating at 400 MHz (Bruker, Karlsruhe, Germany). Tetramethylsilane (TMS) was used as the internal standard for reporting chemical shifts. The frequencies for ^1^H, ^13^C, and DEPT were 400, 125, and 135 MHz, respectively. Metabolites were identified based on their chemical shifts (ppm). Data processing was carried out using software version 14.2. A coupled liquid chromatography system with single quadrupole mass spectrometry and time of flight capabilities was employed for high-resolution ESI-MS measurements (HPLC-EM-SQ-TOF Model G6530BA, Agilent Technologies, Inc., Santa Clara, CA, USA). An AccuTOF Mass Spectrometer (HR-DART-MS) model JMS-T100LC (JEOL Ltd., Peabody, MA, USA) was used to obtain HR-MS data. ECD data were collected using a Circular Dichroism Spectrophotometer J-1500 (JASCO, Oklahoma City, OK, USA). Analytical and preparative high-performance liquid chromatography (HPLC) analyses were conducted using an Agilent 1260 Infinity system (Agilent Technologies Inc., Santa Clara, CA, USA). The setup included a G1311B quaternary pump, a G1367E autosampler, and a G1315C diode array detector (DAD). Analytical HPLC was performed with a Luna Omega Polar C_18_ column (50 × 2.1 mm id, 1.6 µm) from Phenomenex, Inc. (Torrance, CA, USA), while semi-preparative HPLC utilized a Nucleosil C_18_ column (250 × 10 mm id, 5 µm) from Macherey-Nagel, Düren, Germany. Column chromatography was conducted using silica gel (70–230 mesh, Merck, Mexico City, Mexico) or Sephadex LH-20 (Sigma-Aldrich, St. Louis, MO, USA). Thin-layer chromatography was performed on silica gel 60 F254 plates (Macherey-Nagel, Düren, Germany), with visualization achieved using a 10% ceric sulfate solution in H_2_SO_4_ as the color reagent.

### 4.3. Extraction

The plant’s aerial parts were dried in an oven at 40 °C. Two types of extracts were obtained from the plant: an aqueous extract (AE), prepared as an infusion, and an ethanol–water extract (1:1) (EWE), aimed at enhancing compound concentrations and diversity. This setup allowed for a comparative analysis of the profiles and concentrations of compounds in both extracts. The AE was prepared by boiling 500 mL of water, turning off the heat, and adding 20 g of finely ground plant material. The mixture was stirred for 15 min, then filtered and lyophilized. For the EWE preparation, 20 g of finely ground plant material was combined with 500 mL of a 1:1 ethanol–water mixture and heated at 40 °C for four hours, with the process repeated twice. Following filtration, ethanol was removed using a rotary vacuum evaporator (Büchi Labortechnik AG, Flawil, Switzerland), and the remaining concentrated extract was lyophilized. The final yields of the extracts were 3.973 g of AE (19.9%) and 3.796 g of EWE (19%).

For in vivo assays, the traditional human dose was converted to an animal-equivalent dose using a 70 kg person as a model. The formula used was:

Traditional human dose (mg/kg) = crude extraction yield (mg)/70 kg b.w.

To determine the equivalent dose for animals, the human dose was divided by 0.156, a conversion factor for body surface area that corresponds to the body surface area of a 250 g rat and a 70 kg human, as established by the U.S. Food and Drug Administration.

A phytochemical analysis was conducted using 80 g of finely ground plant material, which was extracted in 2000 mL of water following the same method as for the in vivo test. The mixture was then filtered (IE) and extracted with the same volume of ethyl acetate, macerating for three days, resulting in 609 mg of the ethyl acetate soluble fraction (ES) and 17.263 g of the water-soluble fraction (WS).

### 4.4. HPLC/DAD Profiles

To prepare the plant samples (AE, EWE, and isolated compounds), 10.0 mg of AE and EWE were dissolved in 1 mL of a 1:1 mixture of acetonitrile and water. In contrast,1 mg of the isolated compounds was dissolved in the appropriate solvent based on their solubility, which could be acetonitrile, methanol, water, or ethanol. Analyses were conducted at 35 °C with a 2.0 μL injection volume and a flow rate of 0.35 mL/min. Gradient elution was performed using water (with 0.1% formic acid) as solvent A and acetonitrile as solvent B, following this sequence: 99:1 (A:B), 80:20 (A:B) at 14 min, 50:50 (A:B) from 14 to 26 min, 70:30 (A:B) from 26 to 34 min, 20:80 (A:B) from 34 to 35 min, and 99:1 (A:B) from 35 to 38 min. UV detection was performed at wavelengths of 205, 240, 254, 280, and 365 nm. Data acquisition, collection, and processing were managed using OpenLAB LC 1260 (Version A. 01. 06. 111) chromatography software.

### 4.5. HPLC Qualitative Analysis

The chromatographic profiles of AE, WS, and several mixed subfractions were developed. Compounds and standards were injected under the same HPLC conditions. To identify compound **5**, the spectrum at the peak apex was compared to the UV reference spectrum using chromatography software (OpenLAB LC 1260, A. 01. 06. 111). The software calculates a match factor for both spectra, with a high match factor—particularly one close to 1000—indicating a strong similarity between the two compounds [49].

### 4.6. Compounds Isolation

The ES (609 mg) was subjected to a Sephadex LH-20 (Sigma-Aldrich, St. Louis, MO, USA) column and eluted with 100% MeOH, resulting in 24 subfractions (ES1–ES24). Subfractions ES19–ES24 were identified as a pure red oily compound (2.4 mg, **1**). ESF6 and ESF7 (147.2 mg) were combined and purified using column chromatography (14 cm × 3 cm) on silica gel (70–230 mesh, Merck, Mexico City Mexico). The column was eluted sequentially with a mixture of dichloromethane and hexane (50:50), followed by 100% dichloromethane, and then a gradient of dichloromethane and methanol (90:10 to 30:70), yielding 72 collections, which were analyzed by TLC. Similar collections were combined; specifically, collections 31 and 32 were mixed and eluted by preparative TLC using dichloromethane and methanol (95:5), resulting in a yellow oily substance (25.6 mg, **2**). ESF9 and ESF10 (33.1 mg) were combined and separated by preparative TLC with ethyl acetate and methanol (90:10), yielding another yellow oily substance (11.6 mg, **3**). Additionally, 2 g of WS was dissolved in 20 mL of a methanol and water mixture (80:20). After 10 min of sonication and partial filtration, the extract was eluted with 80:20 methanol and water using Sephadex LH-20, resulting in 38 subfractions (WS1–WS38). WS20-WS26 (72.4 mg) were further purified by semi-preparative HPLC. The mobile phase A consisted of water with 0.1% formic acid, while mobile phase B was acetonitrile. A gradient-elution program was applied for sample separation as follows: 0–3 min, 80:20 (A:B); 3–6 min, 75:25 (A:B); 6–7 min, 60:40 (A:B); 7–8 min, 0:100 (A:B); and 8–9 min. The flow rate and detection wavelength were set to 4.72 mL/min and 311 nm, respectively, allowing for the acquisition of 8.5 mg of compound **4** (Rt = 4.65–4.88 min).

Mixtures of WS35 to WS38 (20.2 mg) were analyzed by HPLC against the UV spectrum of standard Luteolin 7-*O*-glucoside (>98% HPLC; Chem Face). The peak at Rt = 14.1 min matched the standard peak completely. The fractions WS35 to WS38 were then eluted by preparative TLC using a solvent mixture of ethyl acetate, methanol, water, and acetic acid (7 mL:3 mL:1 mL:0.5 mL), yielding (3.6 mg, **5**) and (5.1 mg, **6**). Purity criteria for compounds **2**, **3**, and **4** were established prior to in vivo testing using HPLC to ensure that potential impurities would not affect the interpretation of their effects.

### 4.7. Computational Details for Theoretical Circular Dichroism

Compound **2** underwent initial geometry optimization using Spartan ‘14 (Version 1. 1. 4), employing the semiempirical PM3 method to obtain energy-minimized structures. The resulting conformer was screened for redundancy and further refined through density functional theory (DFT) calculations at the B3LYP/DGDZVP level using Gaussian 09 software. This subsequent optimization provided thermochemical parameters, infrared (IR) spectra, and vibrational frequency data at the same level of theory. A time-dependent self-consistent field (TD-SCF) calculation was performed to evaluate the theoretical circular dichroism (TCD) spectra of the conformer in methanol (MeOH), utilizing the default solvent model and the B3LYP/DGDZVP functional. The excitation energies (in nm) and the rotational strengths in terms of dipole velocity (R_vel) were used to simulate the TCD spectra via the Harada–Nakanishi equation, implemented in SpecDis version 1.71 [50].

### 4.8. Experimental Animals

Wistar rats of both sexes, weighing 180 ± 35 g, were obtained from the bioterium of the School of Sciences at UNAM. In-house guidelines for animal protection were strictly followed during the treatment of the animals [51]. The rats had unrestricted access to pellet diets and water throughout the study. They were housed in a controlled environment with a temperature of 25 ± 5 °C and a relative humidity of 60–65%, under a 12 h light/dark cycle. Ethical approval for the study was granted by the Institutional Animal Care and Use Committee of the School of Sciences at UNAM (approval numbers: PI_09_03_2023_01 and PI_09_03_2023b). Blood samples were collected by cutting the tips of the tails, and glucose levels were measured in duplicate every 30 min using Accu-Chek^®^ Active glucometers.

#### 4.8.1. Acute Oral Toxicity Test

According to OECD Test Guidelines 425 (Up and Down Procedure) [52], five female CD1 mice, aged 8–10 weeks and weighing 28 ± 4 g, were randomly selected for the experiment. In a limit test, a single dose of 2000 mg/kg b.w. of AE extract was administered. Prior to dosing, the mice were fasted for three hours and had free access to water. They were closely monitored for the first hour and then regularly for 14 days for any toxic effects. Several parameters were observed during the test, including general activity levels, irritability, responses to touch, tail grip responses, contortion, reflex straightening, auricular reflex, tremors, convulsions, anesthesia, and patterns of urination, defecation, respiration, and mortality. The toxicity evaluation did not include pure compounds.

#### 4.8.2. Induction of Hyperglycemia

Previous studies [53,54] described a method for inducing hyperglycemia. In summary, after an overnight fast, rats were injected with NA (i.p. 150 mg/kg b.w.). After a 15 min interval, an intravenous injection of STZ, dissolved in 0.1 M acetate buffer at pH 4.5, was administered at a dosage of 65 mg/kg b. w. Hyperglycemia was confirmed one week later by a non-fasting blood glucose level exceeding 300 mg/dL, along with symptoms such as polydipsia and polyuria.

#### 4.8.3. Evaluation of Acute Hypoglycemic Effects

The acute hypoglycemic response of the extract and pure compounds was observed over a 2 h period under both fasting and postprandial conditions. During the fasting phase of the experimental design, rats were deprived of food for 4 h and then divided into eight groups (1–8), each consisting of six rats. The treatments administered were as follows:

Group 1: Normal control group receiving a physiological solution.

Group 2: Hyperglycemic control group also receiving a physiological solution.

Group 3: Hyperglycemic positive control group treated with 5 mg/kg b.w. of glibenclamide (Aurax^®^).

Group 4: Hyperglycemic rats treated with 364 mg/kg b.w. of AE extract.

Group 5: Hyperglycemic rats treated with 348 mg/kg b.w. of EWE extract.

Group 6: Hyperglycemic rats treated with 0.58 mg/kg b.w. of compound **2**.

Group 7: Hyperglycemic rats treated with 0.27 mg/kg b.w. of compound **3**.

Group 8: Hyperglycemic rats treated with 1.7 mg/kg b.w. of compound **4**.

Initially, basal blood glucose levels were assessed, followed by the oral administration of a physiological solution (5 mL/kg b.w.) as a vehicle. Blood glucose levels were monitored every 30 min for two hours. After the 4 h fasting period, each of the eight groups received 2 g/kg b.w. of glucose 5 min after treatment to simulate postprandial conditions. Blood glucose levels were measured at baseline and every 30 min for 2 h following glucose loading. Repaglinide (Cinfa^®^, 1 mg/kg b.w.) was used as a positive control. The antihyperglycemic effect throughout the experiment was evaluated using the area under the curve (AUC). Detailed dose calculations for the extracts and pure compounds are provided in the Section 2.

### 4.9. Statistics

To assess the normality of the in vivo experimental data, the D’Agostino–Pearson Omnibus (K2) test was used. An ordinary one-way analysis of variance was performed, followed by the Tukey multiple comparison test to compare group means. For comparisons involving baseline data, a repeated measures analysis of variance was conducted, utilizing Dunnett’s post hoc tests. When the criteria for equal variance were not met, we applied Welch’s ANOVA, followed by Dunnett’s T3 multiple comparison test. The area under the curve (AUC) was calculated using GraphPad Software, version 9.5.1 (www.graphpad.com). This software integrates the area under the plotted curve by summing the trapezoidal areas between data points. AUC values are reported as (mg/dL) × min ± SEM, with a significance level set at *p* < 0.05 for all experiments. Statistical power analyses for sample size determination were conducted using the methods outlined by Charan and Biswas [55]. Other data are presented as mean ± SEM.

## 5. Conclusions

This study provides the first evidence that traditional doses of *Jefea lantanifolia* extracts can safely reduce both fasting and postprandial hyperglycemia. In streptozotocin-nicotinamide (STZ-NA)-induced hyperglycemic rats, both the aqueous extract (AE) and the ethanol–water extract (EWE) demonstrated comparable glucose-lowering effects, exhibiting acute hypoglycemic and antihyperglycemic properties. Chromatographic purification of AE revealed several bioactive constituents, including four flavonoids, a rare sesquiterpene lactone, and a hydroxycinnamic acid derivative. Notably, the hypoglycemic activities of three of these compounds were characterized for the first time, suggesting their potential roles in mediating the plant’s pharmacological effects. While this study focused on acute glycemic control, further research is needed to assess the long-term therapeutic efficacy of *J. lantanifolia*. Future investigations should explore dose–response relationships and therapeutic ranges across diverse experimental models, carefully evaluating both efficacy and safety at higher concentrations. Further studies should evaluate the toxicity of individual compounds to improve our understanding of the safety of crude extracts and their ethnopharmacological significance. In vitro assays should examine the combined effects of these compounds on fasting and postprandial mechanisms associated with acute hypoglycemic and anti-hyperglycemic outcomes, including the inhibition of α-glucosidase and α-amylase by flavonoids and acarbose. Kinetic analyses should determine the types of inhibition, while in vivo experiments should measure blood glucose levels following the administration of these combinations. Additionally, molecular docking studies should investigate binding affinities at both active and allosteric sites of glucose metabolism enzymes, further clarifying their therapeutic potential.

## Figures and Tables

**Table 1:** ^1^H and ^13^C NMR spectroscopy data of compounds **1**, **3** (δ in ppm, *J* in Hz).

Position	*δ_H_* (1)	*δ_H_* (3)	*δ_C_* (3)
2	-	-	167.5
3	6.51, s	6.62, s	102.8
4	-	-	184.2
5	-	-	157.9
6	6.17, d (2.16)	-	134.3
7	-	-	154.3
8	6.40, d (2.13)	6.96, s	95.8
9	-	-	158.0
10	-	-	107.4
1′	-	-	121.1
2′	7.38, d (2.0)	7.86, d (8.7)	128.2
3′	-	6.86, d (8.8)	118.1
4′	-	-	165.8
5′	6.89, d (9.1)	6.86, d (8.8)	118.1
6′	7.37, m	7.86, d (8.7)	128.2
1″	-	5.13, d (7.35)	102.0
2″	-	3.53, m	77.8
3″	-	3.56, m	71.2
4″	-	3.59, m	74.7
5″	-	3.56, m	73.8
6″	-	3.95, dd (12.13, 2.09) 3.72, dd (12.18, 6.0)	62.5
OHC_3_	-	3.89, s	61.5

Measured at 400 MHz in CD_3_OD.

**Table 2:** ^1^H and ^13^C NMR spectroscopy data of compound **2** (δ in ppm, *J* in Hz).

Position	*δ*_H_	*δ*_C_	HMBC	NOESY
1	4.76, d (4.62)	74.9	C-1′, C-3, C-5	H-2*α* H-2 *β*, H-9*β*, H-14
2	*α* 2.14, m *β* 2.45, m	28.9	C-1, C-4	*α*: H-1, H-2*α*, H-3 *β*:H-1, H-2*α*, H-3, H-9, H-14
3	5.32, br s	119.4	C-15	H-2*α* and *β*, H-15
4	-	132.9	-	-
5	2.73, d (11.93)	44.9	C-6, C-4	H-7, H-15, H-9*α*
6	3.97, dd (11.69, 10.74)	79.1	C-4, C-8, C-11, C-14	H-8, H-14, H-15
7	2.56, tt (10.60, 3.10)	56.8	C-5, C-6, C-11	H-5, H-9*α*
8	4.15, td (10.42, 4.50)	67.1	C-6, C-7, C-11	H-9*β*, H-13*β*, H-14
9	*α* 1.62, m *β* 1.74, dd (12.68, 4.56)	44.5	C-1, C-5, C-7, C-8, C-14	*α*: H-7, H-5, H-9*β* *β*: H-8, H-9*α* H-14
10	-	39.4	-	-
11	-	137.0	-	-
12	-	170.5	-	-
13	*α* 6.16, d (3.38) *β* 5.97, d (3.00)	120.2	C-7, C-11, C-12	a:H-13b b:H-8, H-13a
14	0.96, s	18.5	C-1, C-10, C-9	H-1, H-2*β*, H-6, H-8, H-9*β*, H-15
15	1.90, t (1.93)	23.8	C-3, C-4, C-5	H-3, H-6, H-14
1′	-	165.9	-	-
2′	-	139.6	-	-
3′	4.33, br s	62.5	C-1′, C-2′, C-4′	H-4′
4′	*α* 6.24, d (1.14) *β* 5.86, d (1.31)	126.5	C-1′, C-2′, C-3′	H-3′

Measured at 400 MHz in CDCl_3._

**Table 3:** ^1^H and ^13^C NMR spectroscopy data of compound **4** (δ in ppm, *J* in Hz).

Position	δH	δC
1	-	82.6
2	2.33 *α*, dd (16.48, 2.87)/3.05 *β*, dt (16.37, 2.60)	30.7
3	5.27, m	73.3
4	3.80, dd (9.88, 3.62)	73.6
5	4.38, td (11.14, 4.56)	66.9
6	1.79 *α*, dd (13.58, 11.42)/2.46b, dt (13.25, 3.65)	40.8
7	-	177.5
1′, 1″	-	126.6, 126.8
2′, 2″	6.75 d (1.95)	114.6, 114.7
3′,3″	-	143.8
4′, 4″	-	146.8
5′, 5″	6.61, d (8.44); 6.68 d (8.18)	115.6, 115.7
6′, 6″	6.79 d (7.50)	122.5
7′, 7″	7.45 d (15.98); 7.33 d (16.00)	146.8, 146.3
8′, 8″	6.16 d (15.97); 6.27 d (15.97)	114.3, 115.1
9′, 9″	-	169.1, 168.43

Measured at 400 MHz in D_2_O.

**Table 4:** Fasting glucose levels (mg/dL) measured during 2 h acute tests.

Treatments	Dose (mg/kg)	Blood Glucose Levels (mg/dL)				
		0 min	30 min	60 min	90 min	120 min
N	n/a	101 ± 1.5 ^b^ (100%)	101 ± 2 ^b^ (100%)	98 ± 0.7 ^b^ (97%)	100 ± 0.8 ^b^ (99%)	99 ± 1.6 ^b^ (98%)
H	n/a	313 ± 13 (100%)	319 ± 12 (102%)	303 ± 10 (97%)	308 ± 13 (98%)	298 ± 13 (95%)
H + Gli	5	307 ± 5 (100%)	284 ± 7 * (93%)	229 ± 13 ^b,^* (75%)	176 ± 12 ^b,^* (57%)	151 ± 10 ^b,^* (49%)
H + AE	364	303 ± 6 (100%)	298 ± 8 (98%)	252 ± 9 ^b,^* (83%)	236 ± 12 ^b,^* (78%)	203 ± 11 ^b,^* (67%)
H + EWE	348	314 ± 11 (100%)	295 ± 12 * (94%)	260 ± 14 * (83%)	240 ± 13 ^b,^* (76%)	212 ± 12 ^b,^* (68%)
H + compound **2**	0.58	339 ± 4 (100%)	296 ± 16 (87%)	295 ± 7 (87%)	246 ± 10 ^b,^* (73%)	197 ± 11 ^b,^* (58%)
H + compound **3**	0.27	329 ± 12 (100%)	298 ± 11 * (91%)	254 ± 9 * (77%)	217 ± 8 ^b,^* (66%)	198 ± 8 ^b,^* (60%)
H + compound **4**	1.7	329 ± 15 (100%)	296 ± 14 (90%)	257 ± 8 * (78%)	221 ± 4 ^b,^* (67%)	207 ± 4 ^b,^* (63%)

Data are presented as mean ± SEM with *p* < 0.05; n = 6. ^b^ Denotes a statistically significant difference compared to the hyperglycemic control group among different groups. * Denotes a statistically significant difference compared to baseline (T0) within the same group. Group designations are as follows: N (normal control); H (hyperglycemic control); H + Gli (hyperglycemic group treated with Glibenclamide); H + AE (hyperglycemic group treated with aqueous extract of *J. lantanifolia*); H + EWE (hyperglycemic group treated with a 1:1 ethanol–water extract of *J. lantanifolia*); H + compound **2** (hyperglycemic group treated with 2*β*-Hydroxy-dimerostemma brasiolide-1-*O*-(3-hydroxymethacrylate)); H + compound **3** (hyperglycemic group treated with homoplantaginin); H + compound **4** (hyperglycemic group treated with cynarin).

**Table 5:** Postprandial glucose levels (mg/dL) measured during 2 h acute tests.

Treatments	Dose (mg/kg)	Blood Glucose Levels (mg/d*L*)				
		0 min	30 min	60 min	90 min	120 min
N	n/a	109 ± 5 ^b^ (100%)	167 ± 3 ^b,^* (153%)	142 ± 8 ^b,^* (130%)	120 ± 5 ^b^ (110%)	111 ± 3 ^b^ (102%)
H	n/a	298 ± 8 (100%)	521 ± 8 * (175%)	531 ± 12 * (178%)	416 ± 15 * (140%)	406 ± 11 * (136%)
H + Rep	1	312 ± 9 (100%)	350 ± 7 ^b,^* (112%)	310 ± 11 ^b^ (99%)	260 ± 17 ^b,^* (83%)	220 ± 21 ^b,^* (71%)
H + AE	364	324 ± 15 (100%)	456 ± 14 * (141%)	416 ± 16 ^b,^* (128%)	366 ± 9 * (113%)	339 ± 8 (105%)
H + EWE	348	339 ± 8 (100%)	436 ± 14 ^b,^* (129%)	391 ± 13 ^b^ (115%)	366 ± 13 (108%)	342 ± 11 ^b^ (101%)
H + compound **2**	0.58	348 ± 5 (100%)	462 ± 8 ^b,^* (133%)	430 ± 11 ^b,^* (124%)	391 ± 10 * (112%)	354 ± 5 ^b^ (102%)
H + compound **3**	0.27	338 ± 16 (100%)	441 ± 20 * (130%)	411 ± 9 ^b,^* (122%)	369 ± 14 * (109%)	332 ± 17 (98%)
H + compound **4**	1.7	325 ± 15 (100%)	452 ± 9 ^b,^* (139%)	423 ± 4 ^b,^* (130%)	398 ± 5 (122%)	375 ± 9 (115%)

Data are presented as mean ± SEM with *p* < 0.05; n = 6. ^b^ Denotes a statistically significant difference compared to the hyperglycemic control group among different groups. * Denotes a statistically significant difference compared to baseline (T0) within the same group. Group designations are as follows: N (normal control); H (hyperglycemic control); H + Rep (hyperglycemic group treated with Repaglinide); H + AE (hyperglycemic group treated with aqueous extract of *J. lantanifolia*); H + EWE (hyperglycemic group treated with a 1:1 ethanol–water extract of *J. lantanifolia*); H + compound **2** (hyperglycemic group treated with 2*β*-Hydroxy-dimerostemma brasiolide-1-*O*-(3-hydroxymethacrylate)); H + compound **3** (hyperglycemic group treated with homoplantaginin); H + compound **4** (hyperglycemic group treated with cynarin).

## Data Availability

The original contributions presented in this study are included in the article/Supplementary Material. Further inquiries can be directed at the corresponding author.

## Supplementary Materials

The following supporting information can be downloaded at: https://www.mdpi.com/article/10.3390/plants14193054/s1, Figure S1: HRESI-MS spectrum of compound **2**; Figure S2: ^1^H-NMR spectrum of compound **2** (400 MHz, CDCl_3_); Figure S3: ^13^C-NMR spectrum of compound **2** (125 MHz, CDCl_3_); Figure S4: DEPT spectrum of compound **2** (135 MHz, CDCl_3_); Figure S5: HSQC spectrum of compound **2** (400 MHz, CDCl_3_); Figure S6: COSY spectrum of compound **2** (400 MHz, CDCl_3_); Figure S7: HMBC spectrum of compound **2** (400 MHz, CDCl_3_); Figure S8: NOESY spectrum of compound **2** (400 MHz, CDCl_3_); Figure S9: TOCSY spectrum of compound **2** (400 MHz, CDCl_3_); Figure S10: HRESI-MS spectrum of compound **3**; Figure S11: ^1^H-NMR spectrum of compound **3** (400 MHz, CD_3_OD); Figure S12: ^13^C-NMR spectrum of compound **3** (125 MHz, CD_3_OD); Figure S13: COSY spectrum of compound **3** (400 MHz, CD_3_OD); Figure S14: HSQC spectrum of compound **3** (400 MHz, CD_3_OD); Figure S15: HMBC spectrum of compound **3** (400 MHz, CD_3_OD); Figure S16: NOESY spectrum of compound **3** (400 MHz, CD_3_OD); Figure S17: HRESI-MS spectrum of compound **6**; Figure S18: HRESI-MS spectrum of compound **4**; Figure S19: ^1^H-NMR spectrum of compound **4** (400 MHz, D_2_O); Figure S20: ^13^C-NMR spectrum of compound **4** (125 MHz, D_2_O); Figure S21: DEPT-NMR spectrum of compound **4** (135 MHz, D_2_O); Figure S22: COSY-NMR spectrum of compound **4** (400 MHz, D_2_O); Figure S23: HMBC-NMR spectrum of compound **4** (400 MHz, D_2_O); Figure S24: HRESI-MS spectrum of compound **1**.

## Abbreviations

The following abbreviations are used in this manuscript:

STZ	streptozotocin
NA	nicotinamide
T2D	type 2 diabetes
i.p.	intraperitoneally
