# Supplementary material for: Phytochemical Composition and Acute Hypoglycemic Effect of Jefea lantanifolia (S. Schauer) Strother in Rats
Source: Plants (Basel). 2025 Oct 2;14(19):3054. doi: 10.3390/plants14193054 (PMC12526190; doi:10.3390/plants14193054)
Supplement: Supplementary file 1 [file plants-14-03054-s001.zip › plants-3810654-supplementary.pdf]

SUPPLEMENTARY MATERIAL

Assessing the Acute Hypoglycemic Effects of *Jefea lantanifolia* (S. Schauer) Strother and Its Compounds in Rats

Fereshteh Safavi, Sonia M. Escandón-Rivera, Adolfo Andrade-Cetto *
Laboratorio de Etnofarmacología, Departamento de Biología Celular, Facultad de Ciencias, Universidad Nacional Autónoma de México, Av. Universidad 3000, Circuito Exterior S/N, Delegación Coyoacán, C.P, 04510, Ciudad Universitaria, Ciudad de México, México

* Corresponding author.
E-mail addresses: aac@ciencias.unam.mx

Figure S1. HRESI-MS spectrum of compound **2.**

Figure S2. ^{1}H-NMR spectrum of compound **2** (400 MHz, $CDCl_3$).

Figure S3. ^{13}C-NMR spectrum of compound **2** (125 MHz, $CDCl_3$).

Figure S4. DEPT spectrum of compound **2** (135 MHz, $CDCl_3$).

Figure S5. HSQC spectrum of compound **2** (400 MHz, $CDCl_3$).

Figure S6. COSY spectrum of compound **2** (400 MHz, $CDCl_3$).

Figure S7. HMBC spectrum of compound **2** (400 MHz, $CDCl_3$).

Figure S8. NOESY spectrum of compound **2** (400 MHz, $CDCl_3$).

Figure S9. TOCSY spectrum of compound **2** (400 MHz, $CDCl_3$).

Figure S10. HRESI-MS spectrum of compound **3.**

Figure S11. ^{1}H-NMR spectrum of compound **3** (400 MHz, CD_3OD).

Figure S12. ^{13}C-NMR spectrum of compound **3** (125 MHz, CD_3OD).

Figure S13. COSY spectrum of compound **3** (400 MHz, CD_3OD).

Figure S14. HSQC spectrum of compound **3** (400 MHz, CD_3OD).

Figure S15. HMBC spectrum of compound **3** (400 MHz, CD_3OD).

Figure S16. NOESY spectrum of compound **3** (400 MHz, CD_3OD).

Figure S17. HRESI-MS spectrum of compound **6.**

Figure S18. HRESI-MS spectrum of compound **4.**

Figure S19. ^{1}H-NMR spectrum of compound **4** (400 MHz, D_2O).

Figure S20. ^{13}C-NMR spectrum of compound **4** (125 MHz, D_2O).

Figure S21. DEPT-NMR spectrum of compound **4** (135 MHz, D_2O).

Figure S22. COSY-NMR spectrum of compound **4** (400 MHz, D_2O).

Figure S23. HMBC-NMR spectrum of compound **4** (400 MHz, D_2O).

Figure S24. HRESI-MS spectrum of compound **1**.

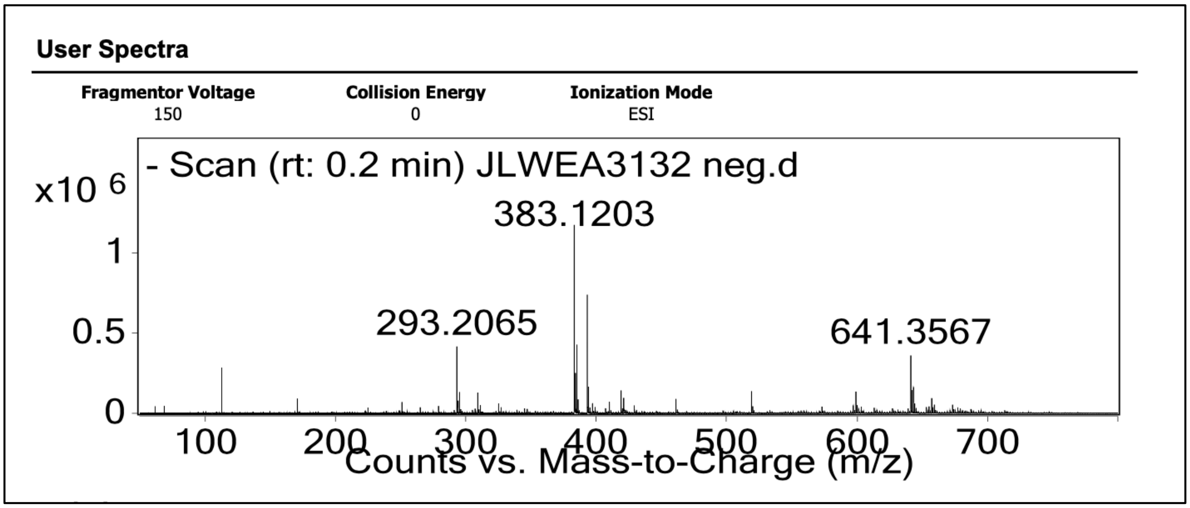

Figure S1. HRESI-MS spectrum of compound **2**

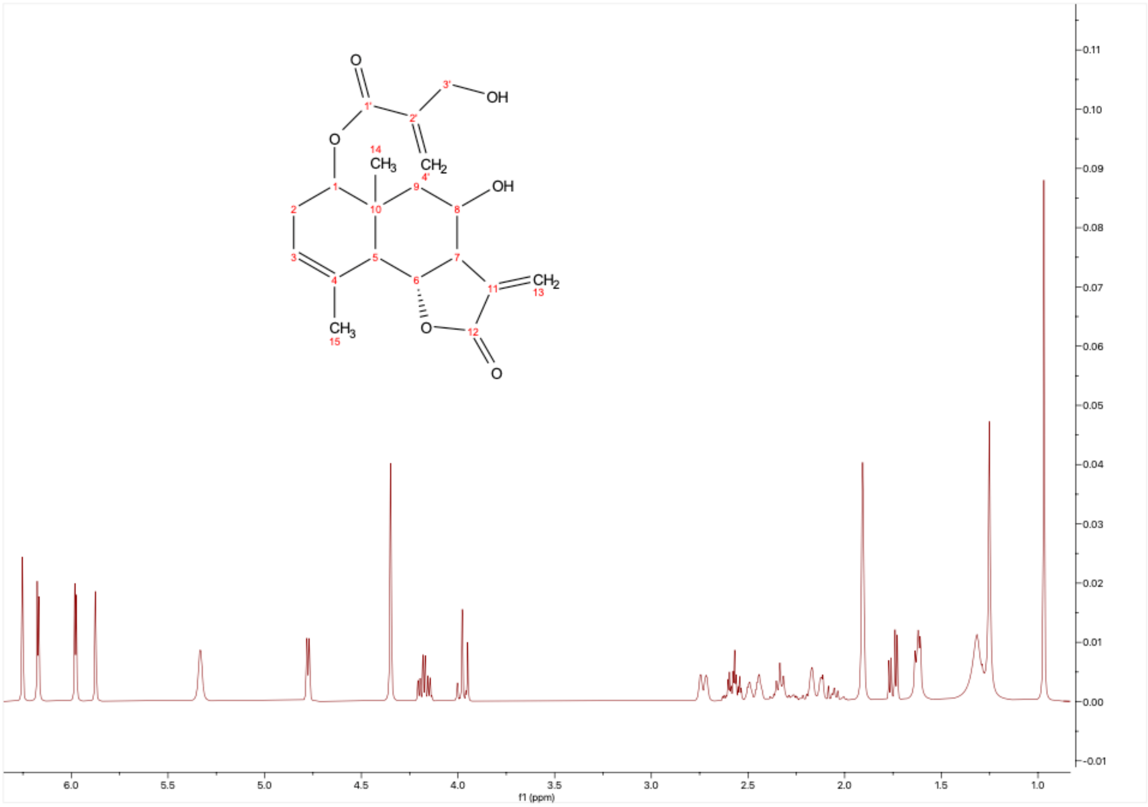

Figure S2. ^{1}H-NMR spectrum of compound **2** (400 MHz, $CDCl_3$).

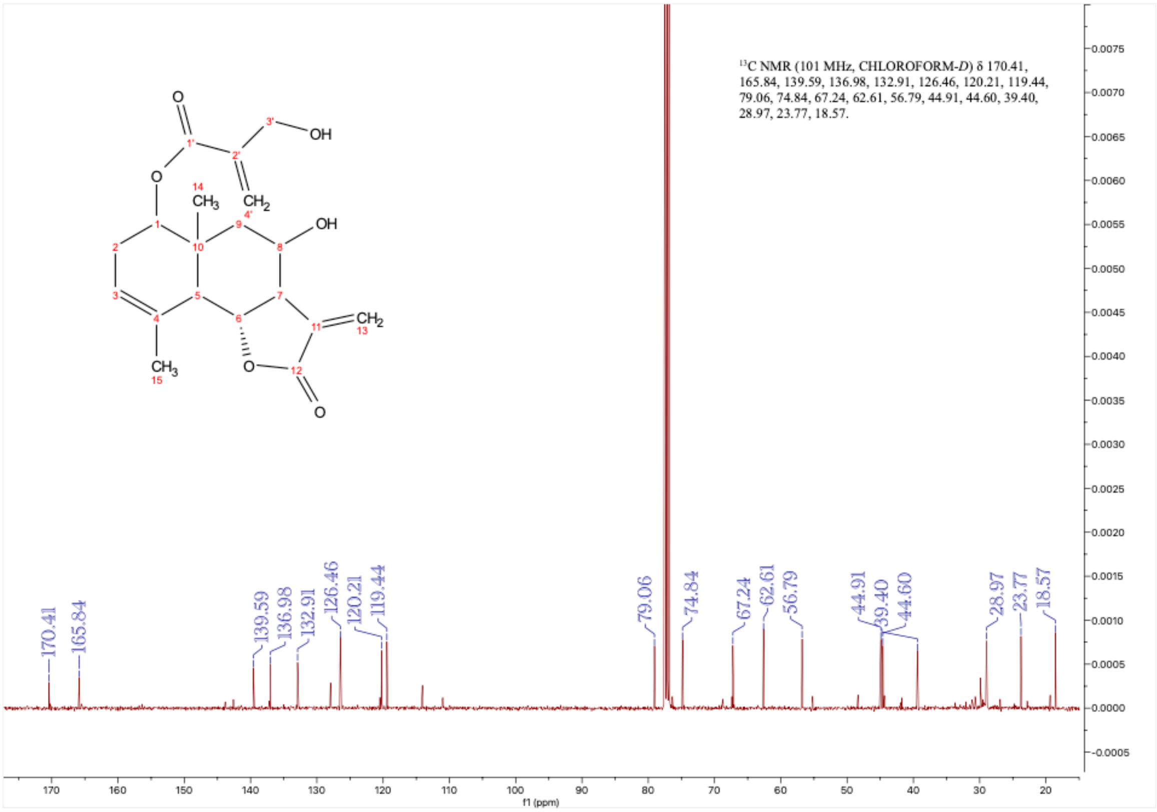

Figure S3. ^{13}C-NMR spectrum of compound **2** (125 MHz, $CDCl_3$).

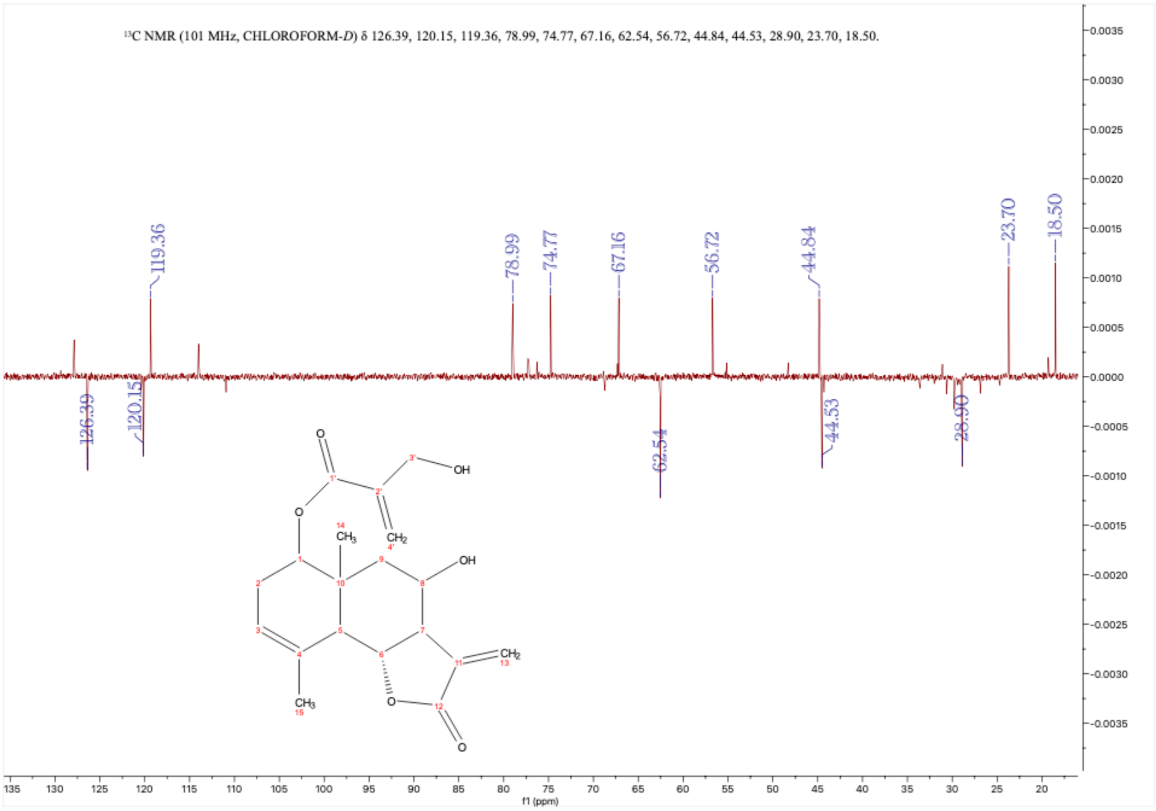

Figure S4. DEPT spectrum of compound **2** (135 MHz, $CDCl_3$).

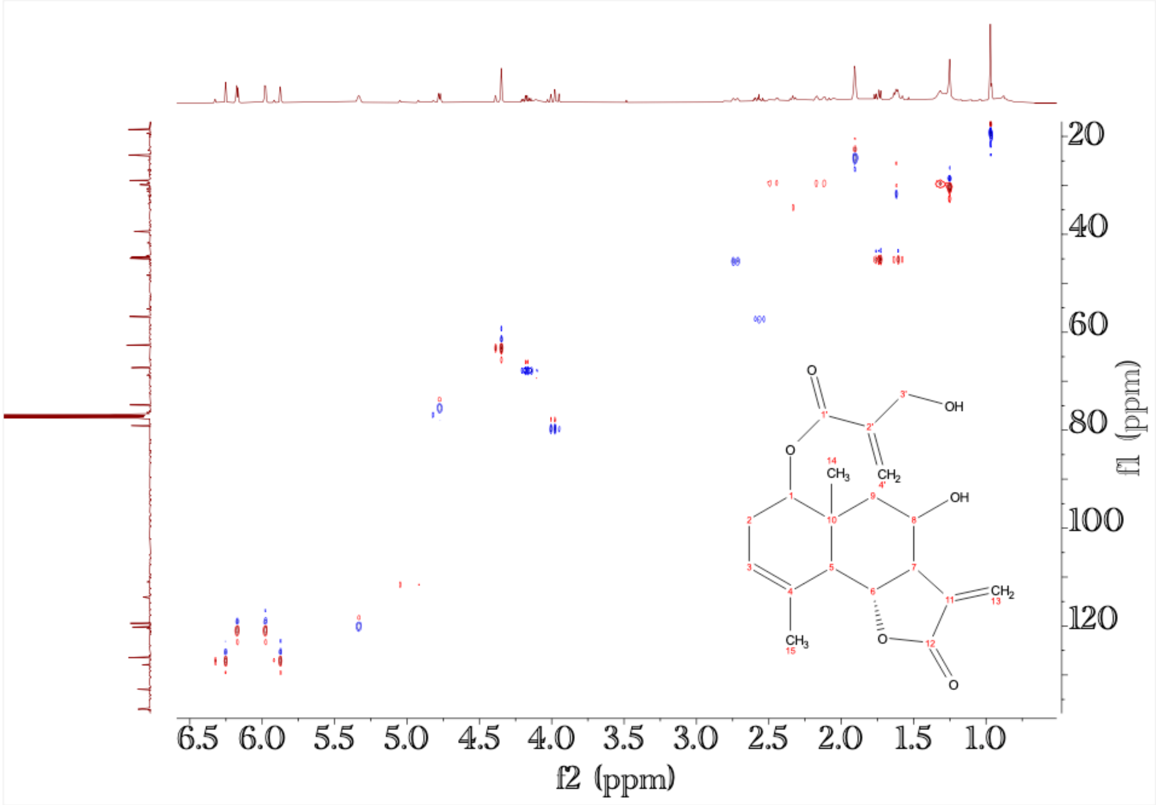

Figure S5. HSQC spectrum of compound **2** (400 MHz, $CDCl_3$).

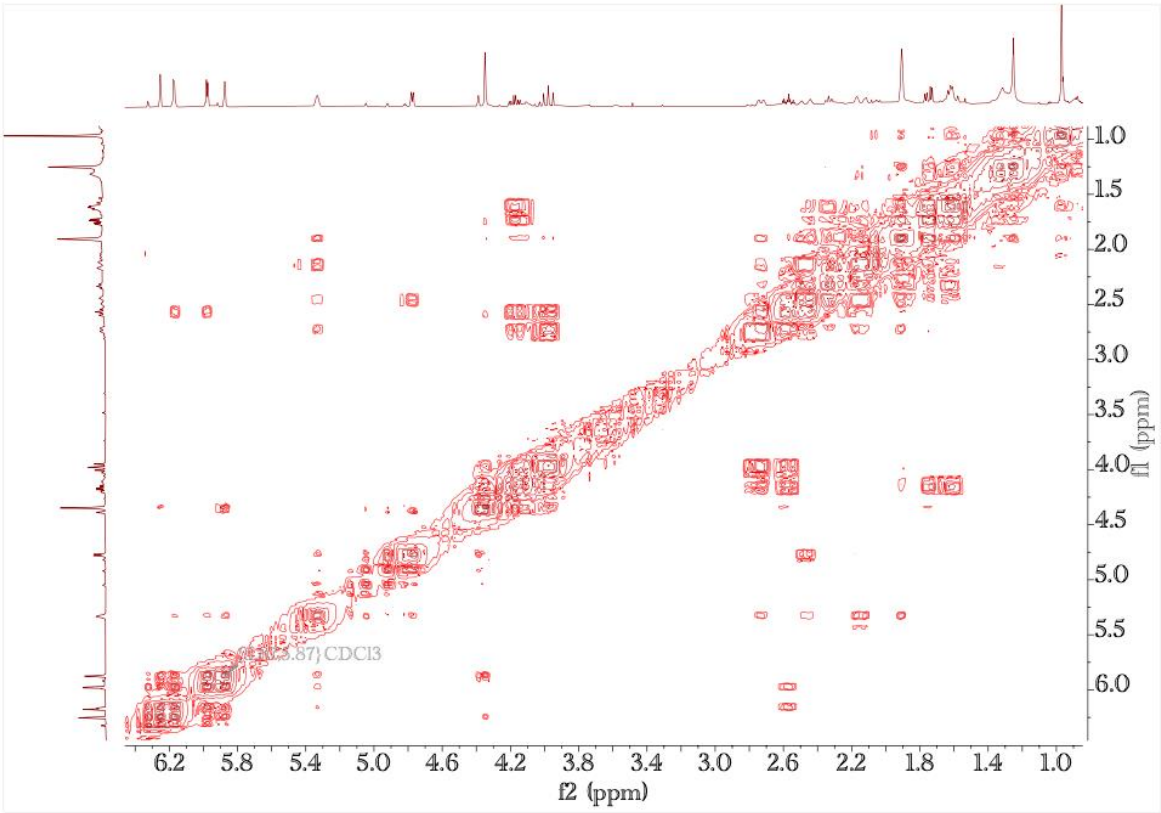

Figure S6. COSY spectrum of compound **2** (400 MHz, $CDCl_3$).

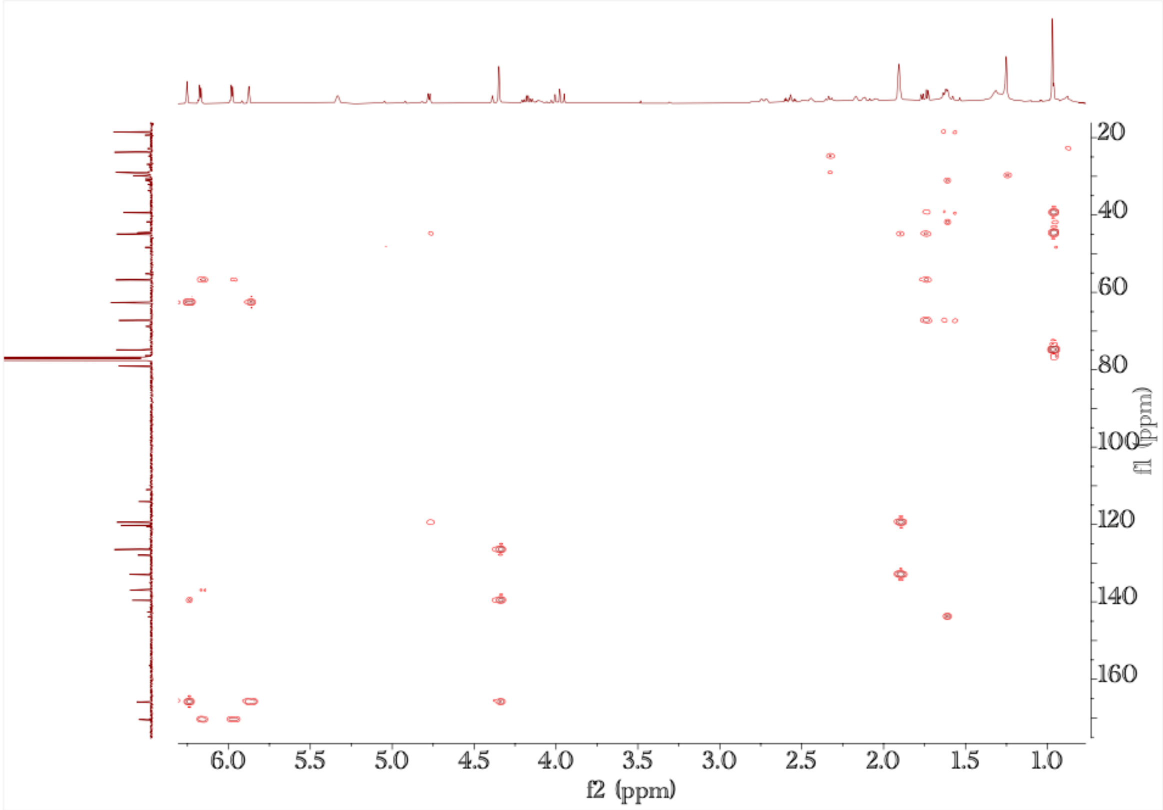

Figure S7. HMBC spectrum of compound **2** (400 MHz, $CDCl_3$).

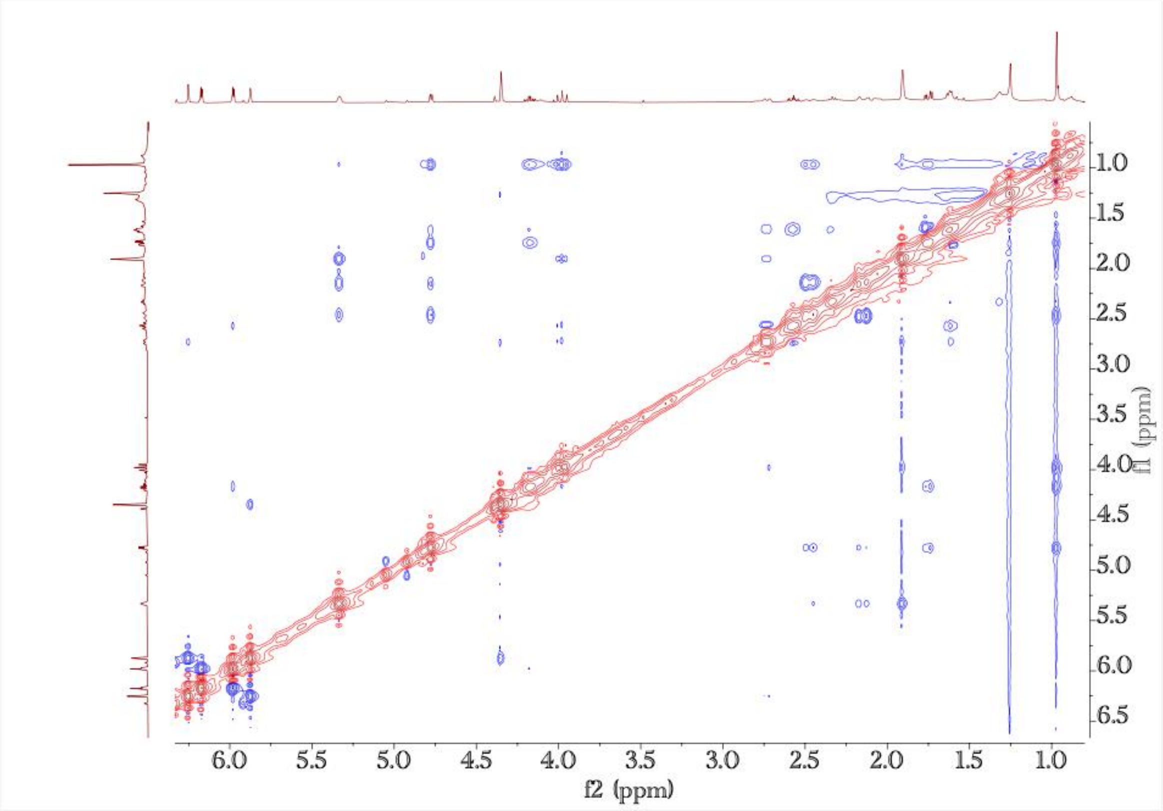

Figure S8. NOESY spectrum of compound **2** (400 MHz, $CDCl_3$).

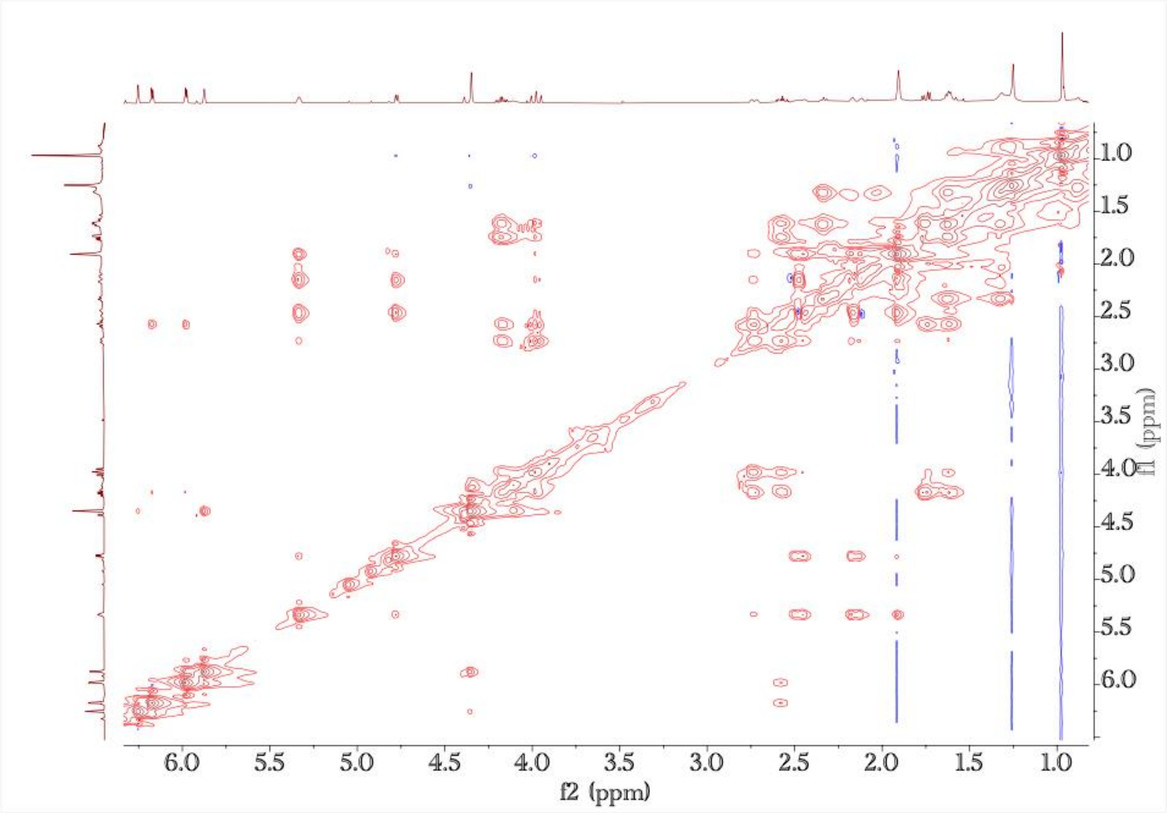

Figure S9. TOCSY spectrum of compound **2** (400 MHz, $CDCl_3$).

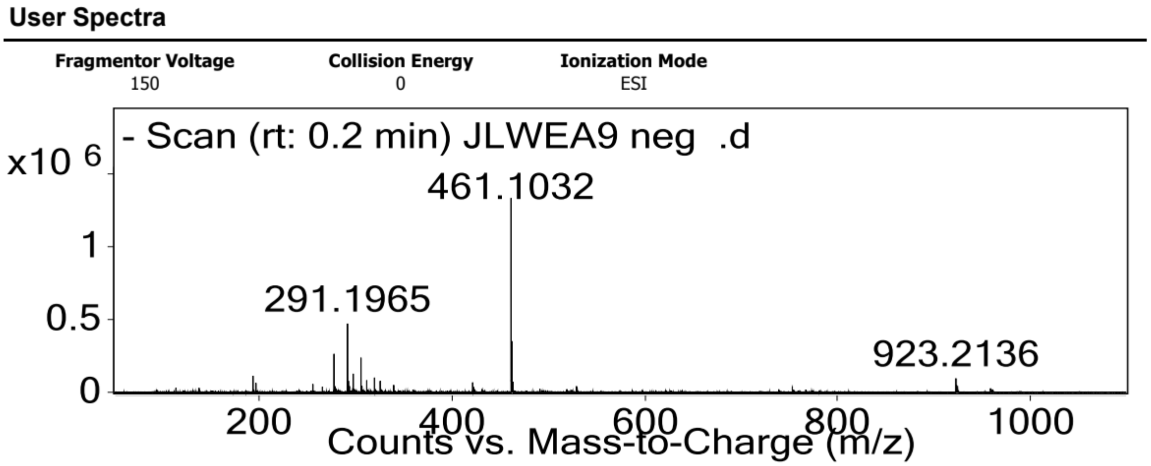

Figure S10. HRESI-MS spectrum of compound **3.**

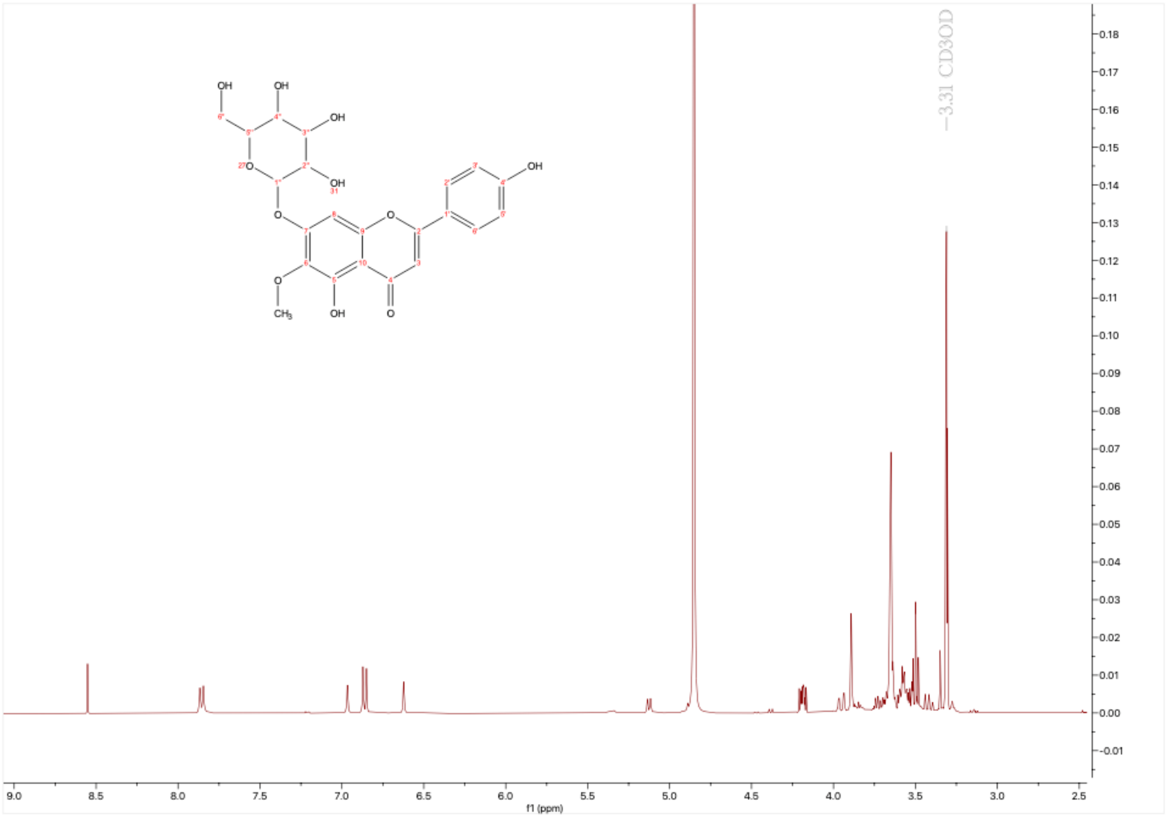

Figure S11. ^{1}H-NMR spectrum of compound **3** (400 MHz, CD_3OD).

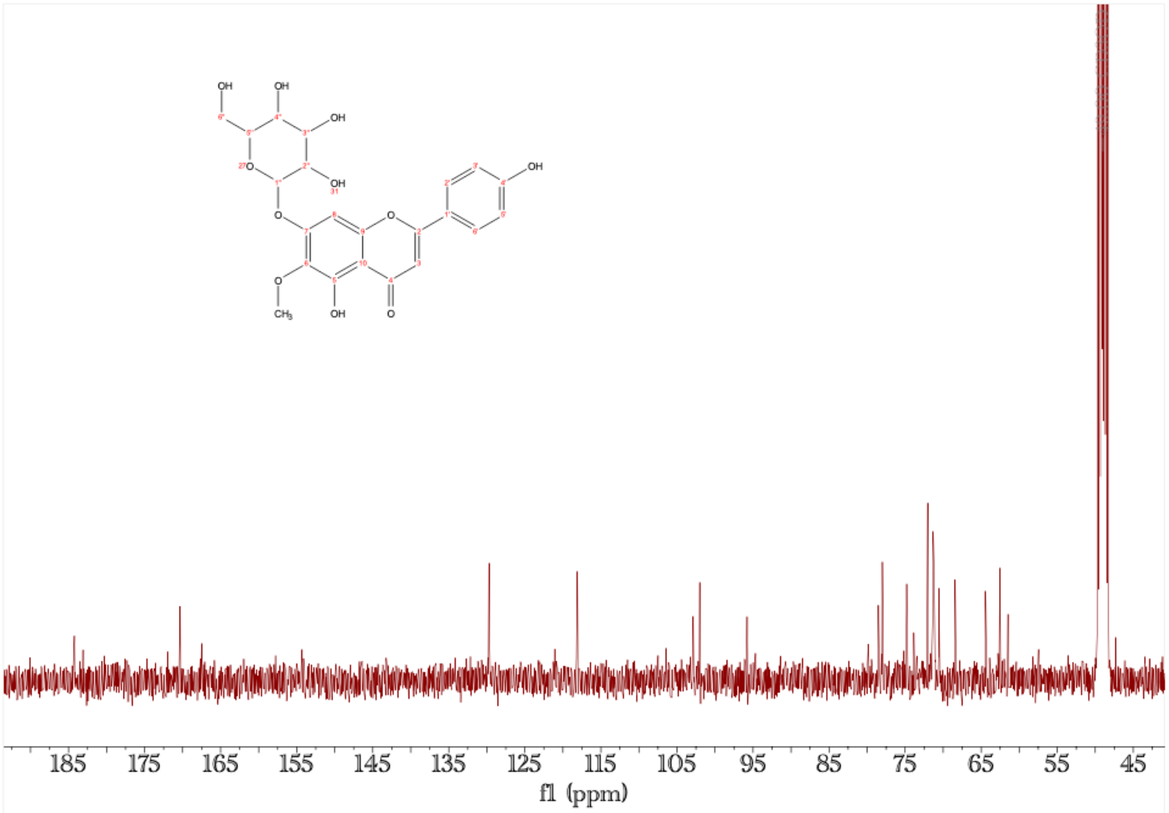

Figure S12. ^{13}C-NMR spectrum of compound **3** (125 MHz, CD_3OD).

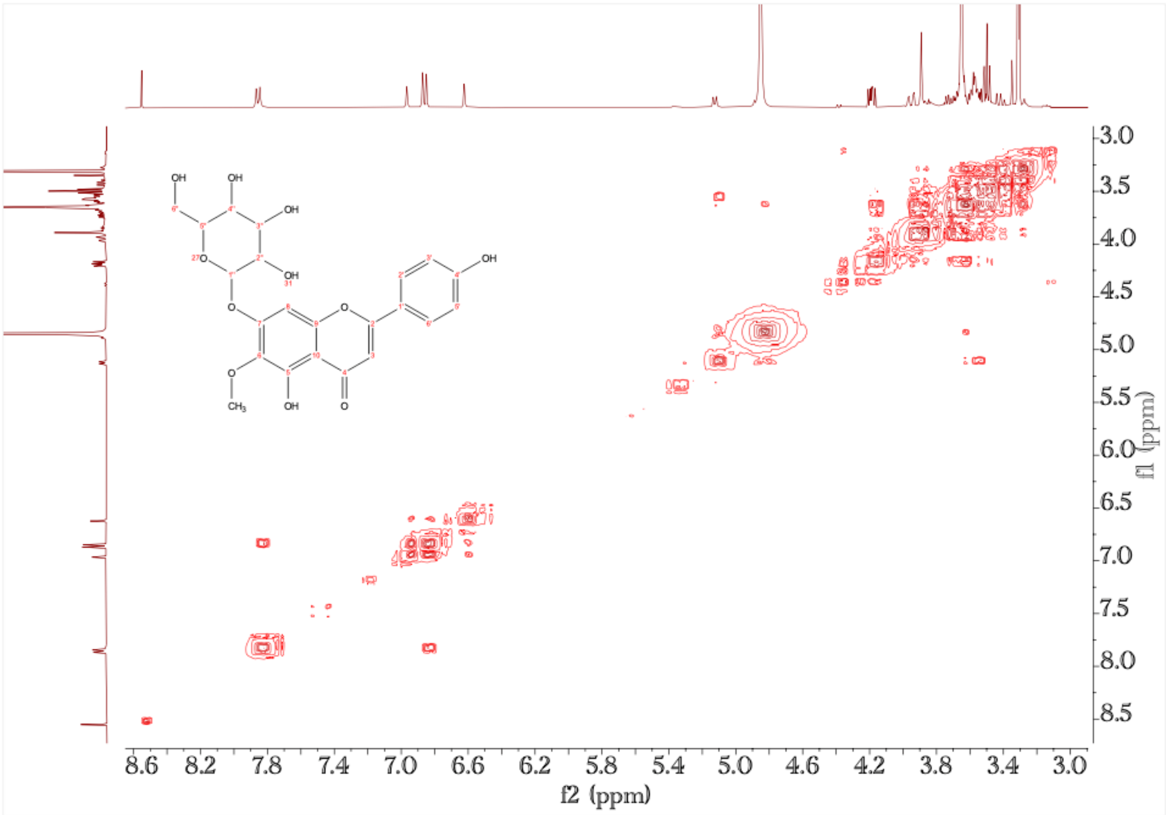

Figure S13. COSY spectrum of compound **3** (400 MHz, CD_3OD).

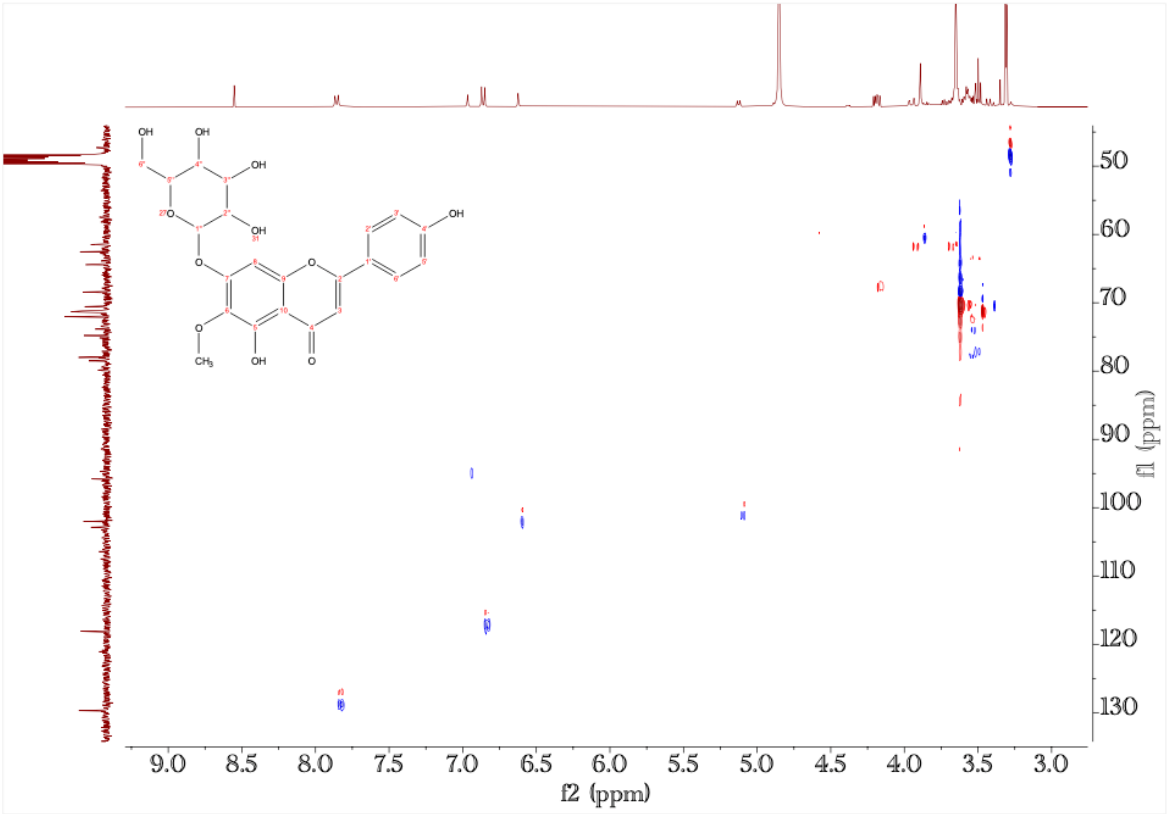

Figure S14. HSQC spectrum of compound **3** (400 MHz, CD_3OD).

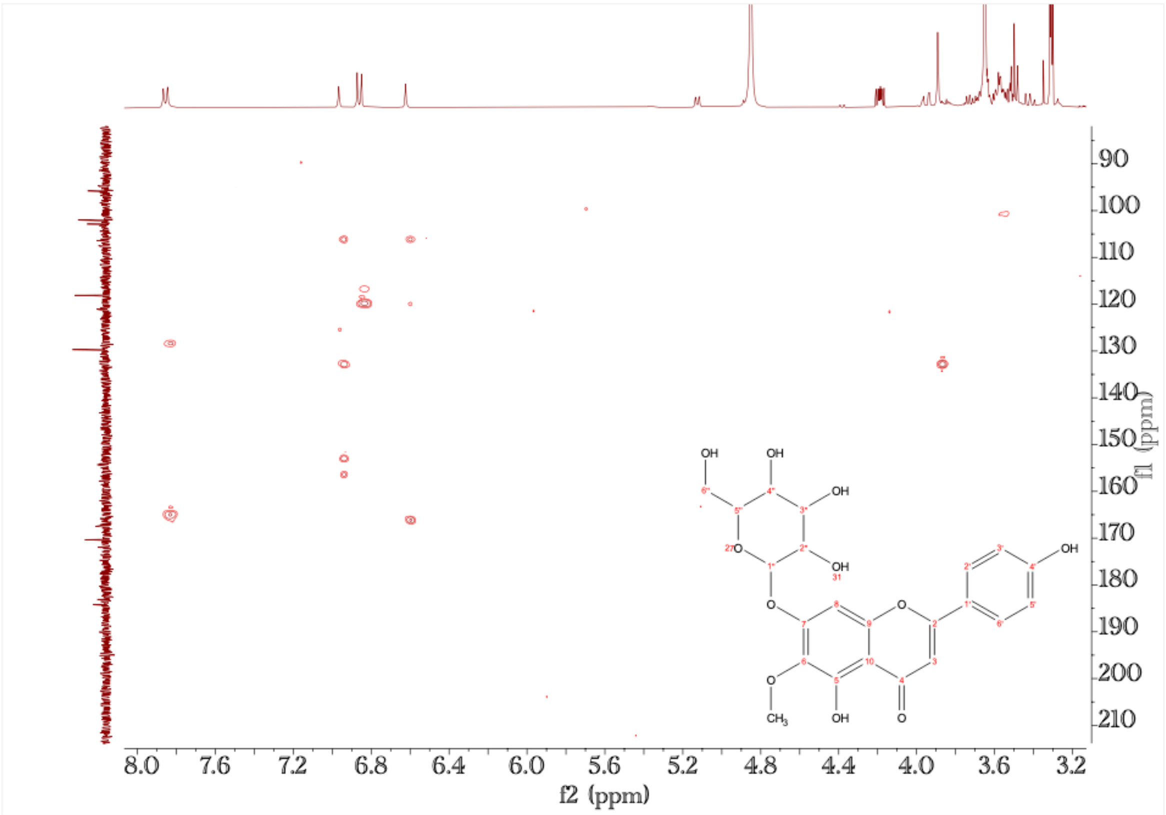

Figure S15. HMBC spectrum of compound **3** (400 MHz, CD_3OD).

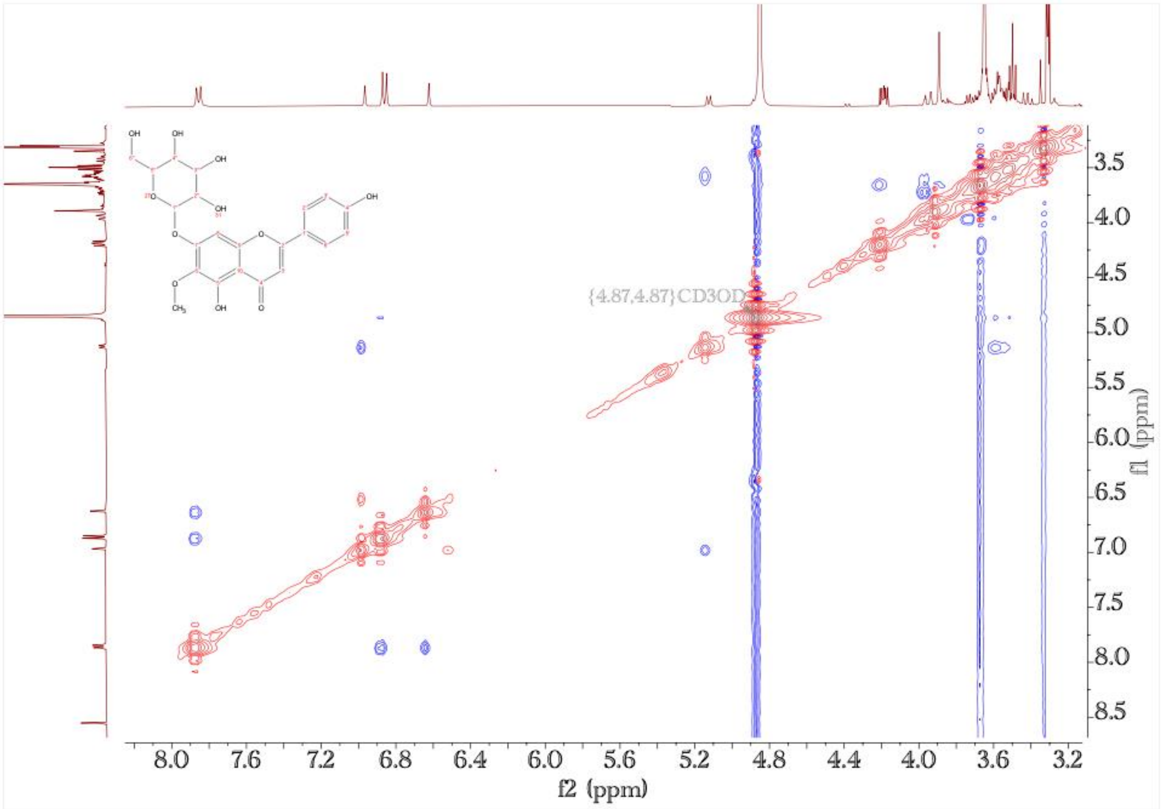

Figure S16. NOESY NMR spectrum of compound **3** (400 MHz, CD_3OD).

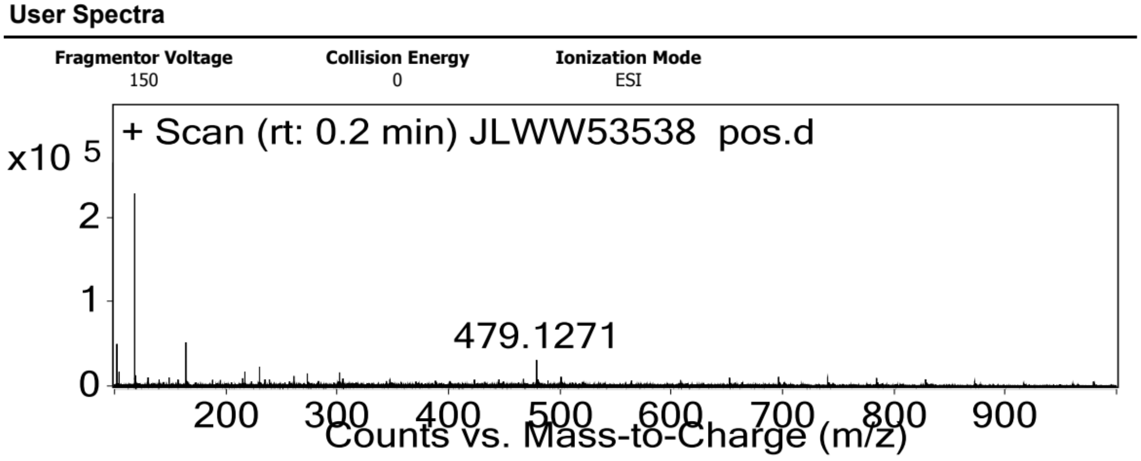

Figure S17. HRESI-MS spectrum of compound **6.**

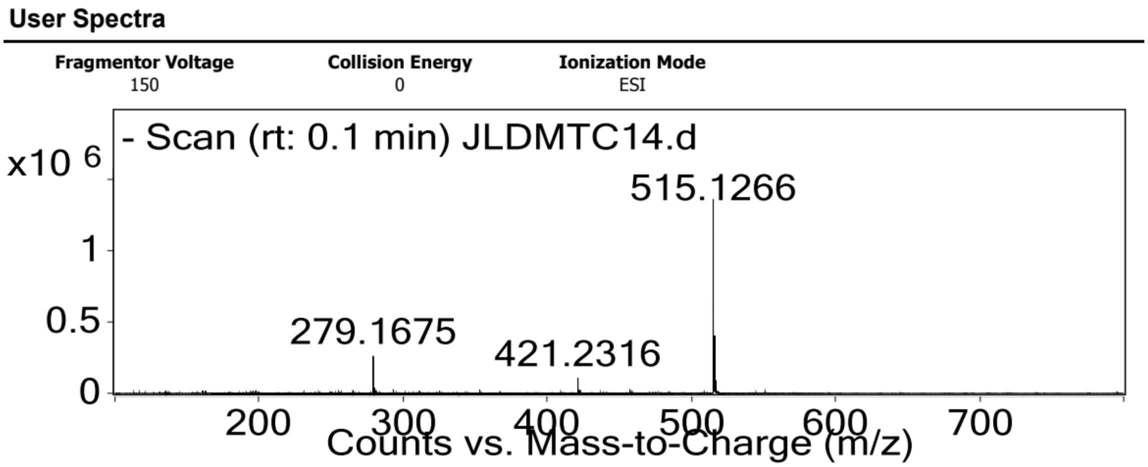

Figure S18. HRESI-MS spectrum of compound **4.**

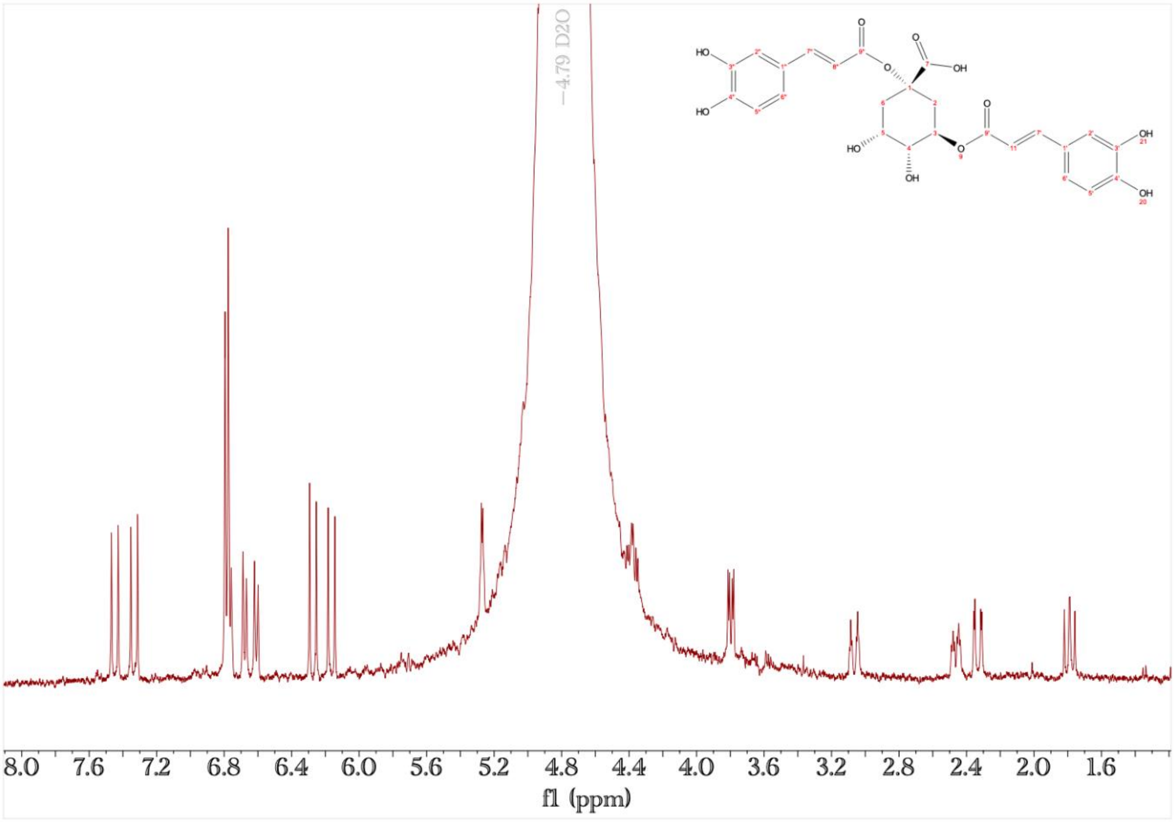

Figure S19. ^{1}H-NMR spectrum of compound **4** (400 MHz, D_2O).

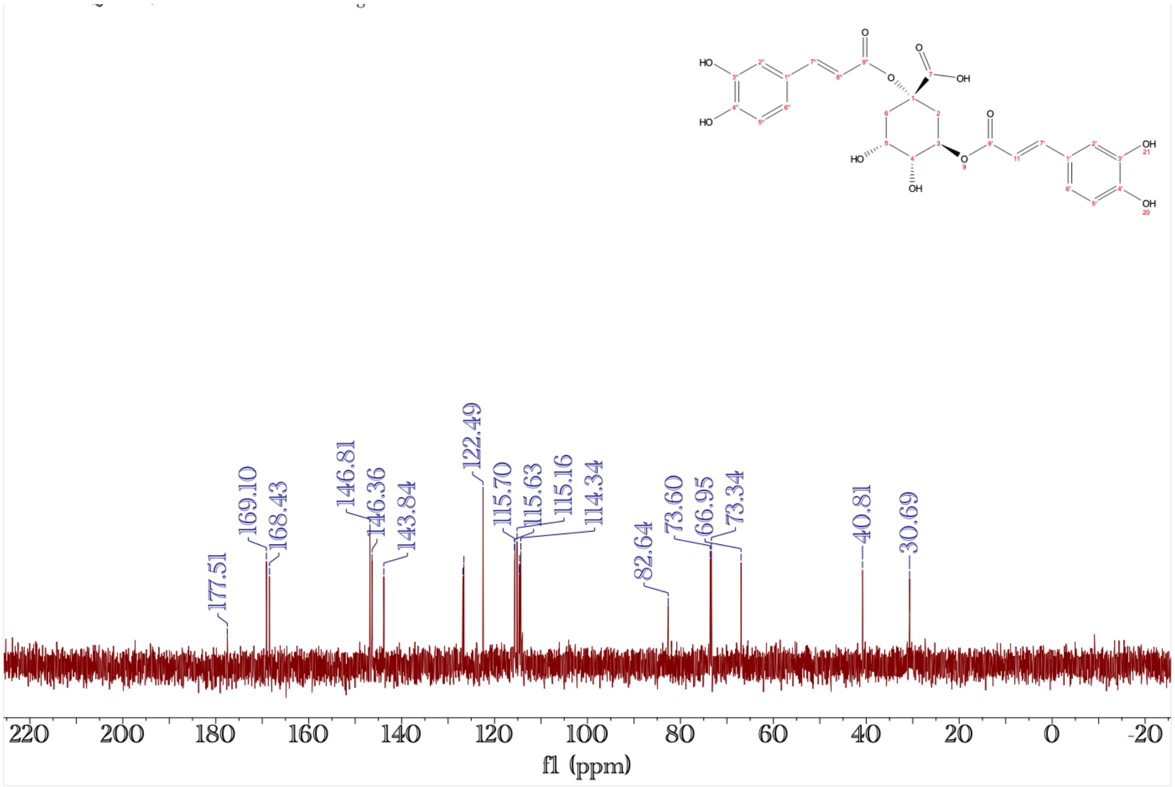

Figure S20. ^{13}C-NMR spectrum of compound **4** (125 MHz, D_2O).

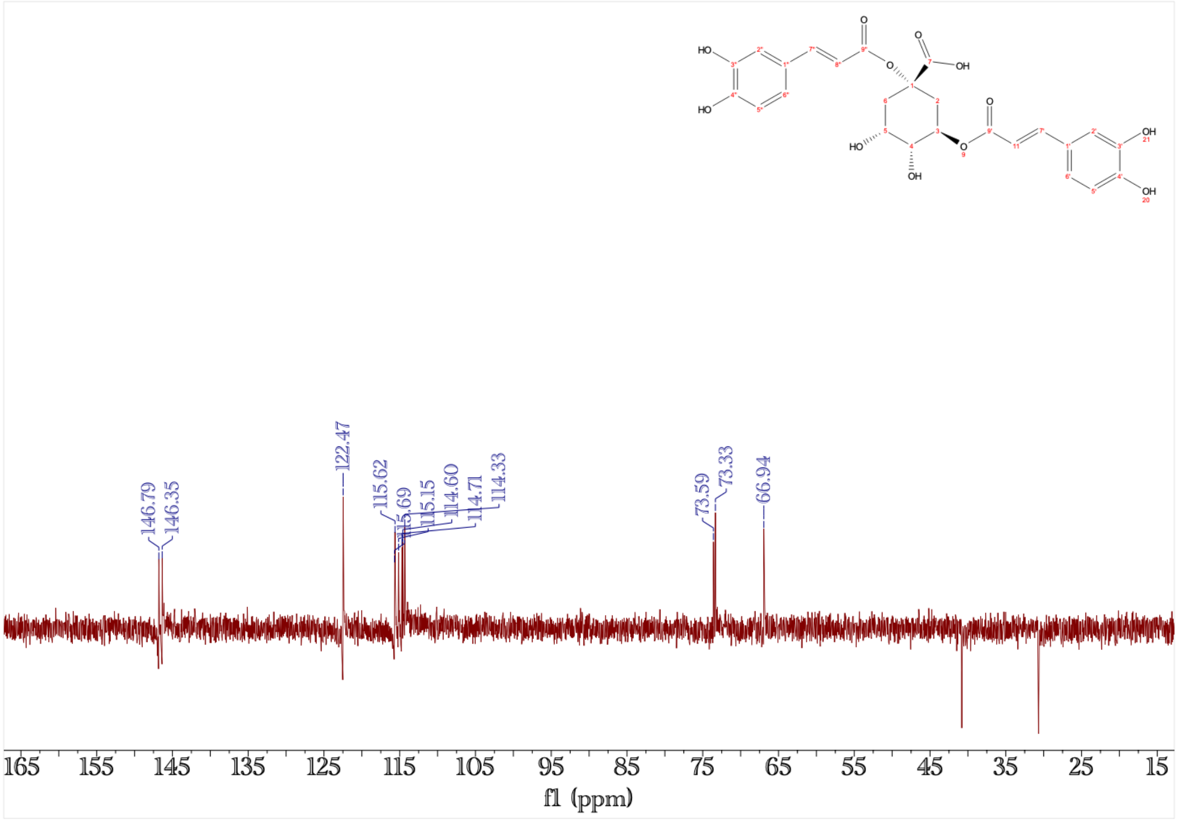

Figure S21. DEPT-NMR spectrum of compound **4** (135 MHz, D_2O).

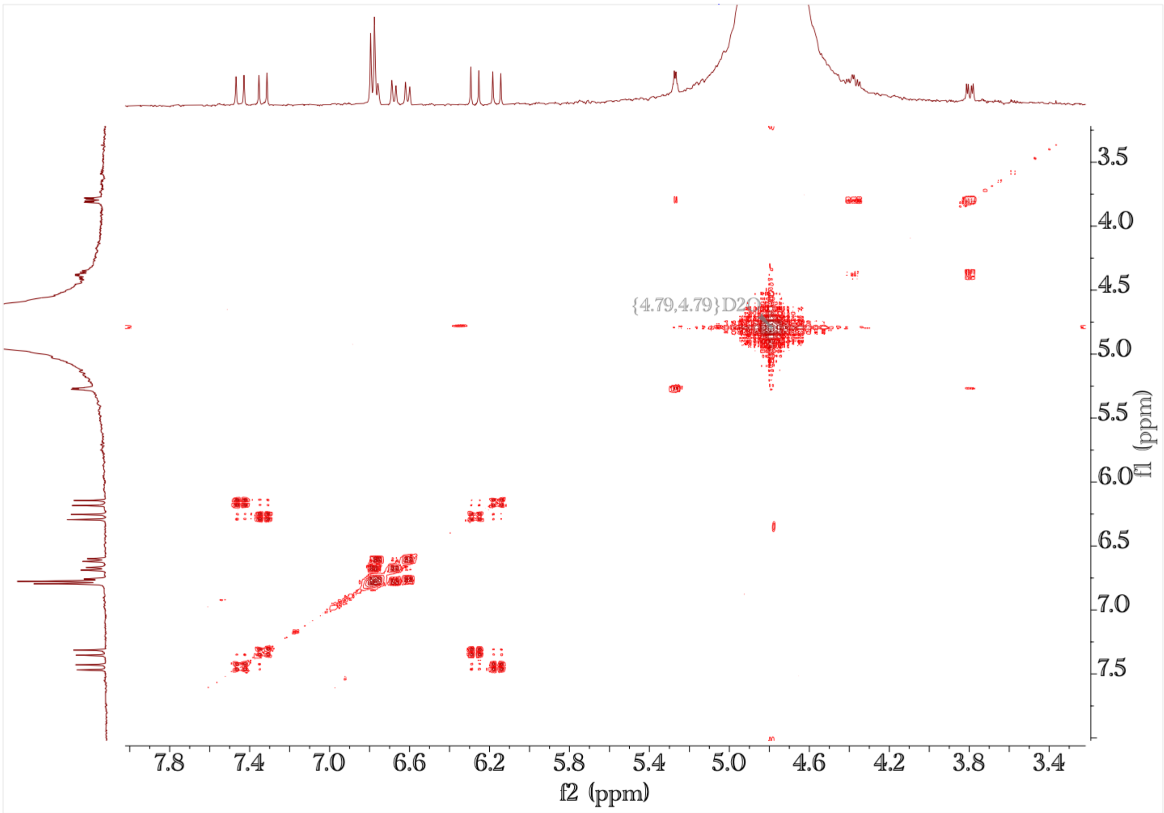

Figure S22. COSY-NMR spectrum of compound **4** (400 MHz, D_2O).

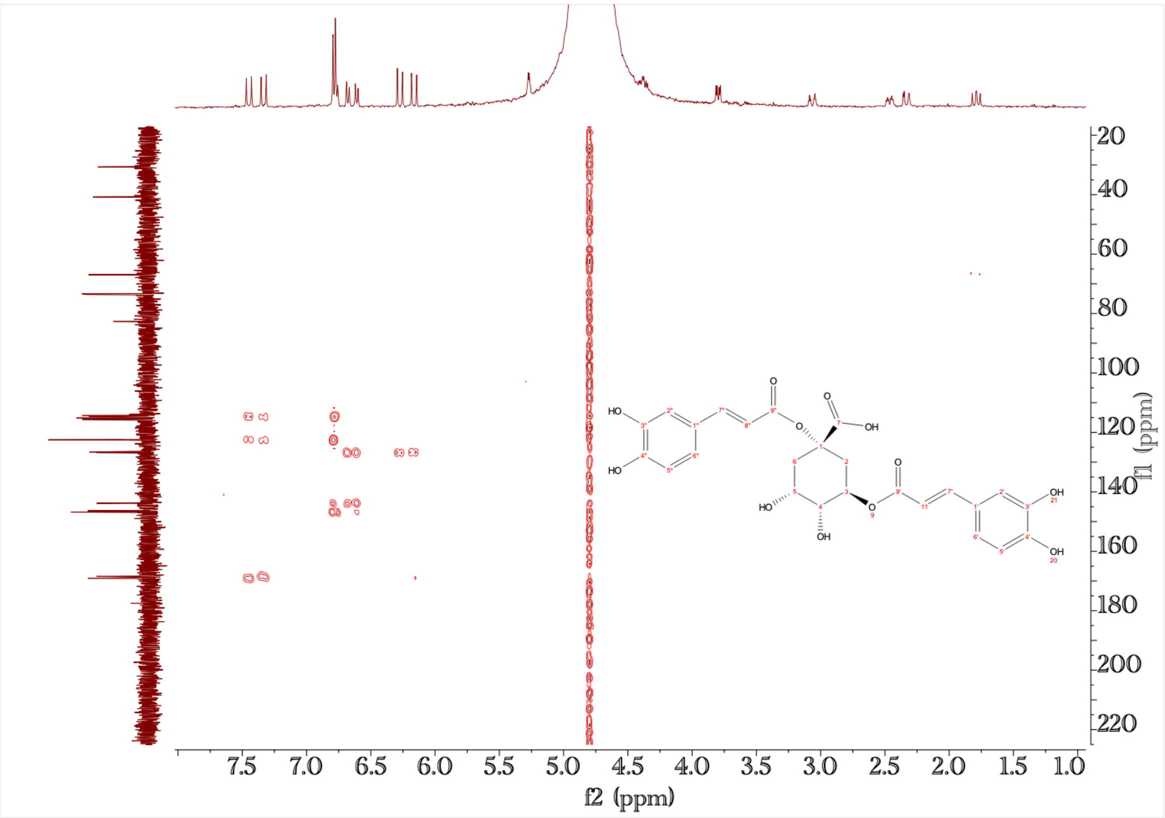

Figure S23. HMBC-NMR spectrum of compound **4** (400 MHz, D_2O).

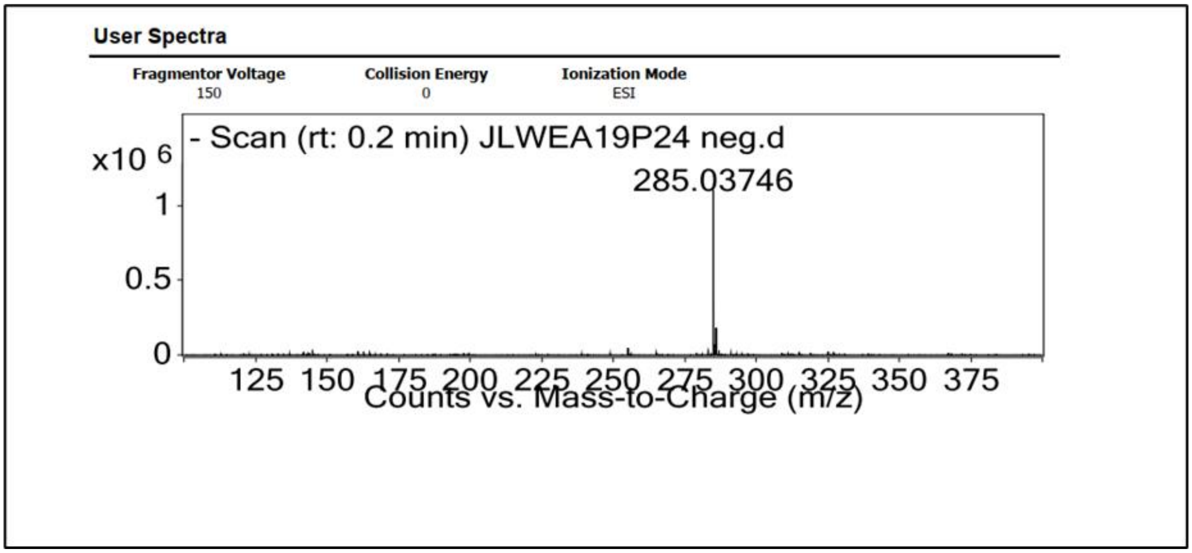

Figure S24. HRESI-MS spectrum of compound **1**.
